# Supplementary material for: Trends and determinants of length of stay and hospital reimbursement following knee and hip replacement: evidence from linked primary care and NHS hospital records from 1997 to 2014
Source: BMJ Open. 2018 Jan 27;8(1):e019146. doi: 10.1136/bmjopen-2017-019146 (PMC5829869; doi:10.1136/bmjopen-2017-019146)
Supplement: Supplementary data [file bmjopen-2017-019146supp001.pdf]

APPENDIX

Table of Contents

Patient characteristics by year of surgery 2

Primary total knee replacement 2

Primary total hip replacement 4

Revision total knee replacement 6

Revision total hip replacement 8

Length of stay by year of surgery 10

Hospital costs by year of surgery 12

Partial effect of age on length of stay and costs 14

Partial effect of age at surgery on length of stay from multivariable regressions. Predicted length of stay by age while other explanatory variables are held fixed at their average (median if continuous and mode if categorical). TKR: total knee replacement, THR: total hip replacement. 14

Partial effect of age at surgery on hospital costs (in 2017 GBP) from multivariable regressions. Predicted cost by age while other explanatory variables are held fixed at their average (median if continuous and mode if categorical). TKR: total knee replacement, THR: total hip replacement. 14

PATIENT CHARACTERISTICS BY YEAR OF SURGERY

Primary total knee replacement

					RCS Charlson score (n (%))				IMD quintiles (n (%))				
Year of surgery	n	Age (mean (sd))	Gender: Male (n (%))	Rheumatoid arthritis (n (%))	0	1	2	3+	1	2	3	4	5
1997	45	72 (9)	18 (40%)	8 (18%)	43 (96%)	2 (4%)	0 (0%)	0 (0%)	7 (16%)	9 (20%)	13 (29%)	8 (18%)	8 (18%)
1998	64	72 (10)	23 (36%)	12 (19%)	57 (89%)	5 (8%)	1 (0%)	1 (0%)	11 (17%)	14 (22%)	16 (25%)	14 (22%)	9 (14%)
1999	114	71 (9)	45 (39%)	6 (5%)	98 (86%)	14 (12%)	2 (0%)	0 (0%)	18 (16%)	32 (28%)	17 (15%)	30 (27%)	16 (14%)
2000	115	72 (9)	55 (48%)	16 (14%)	97 (84%)	16 (14%)	1 (0%)	1 (0%)	22 (19%)	31 (27%)	19 (17%)	21 (18%)	22 (19%)
2001	170	71 (9)	76 (45%)	18 (11%)	152 (89%)	16 (9%)	2 (0%)	0 (0%)	40 (24%)	34 (20%)	43 (25%)	31 (18%)	22 (13%)
2002	268	70 (10)	116 (43%)	28 (10%)	237 (88%)	28 (10%)	3 (0%)	0 (0%)	68 (25%)	67 (25%)	61 (23%)	39 (15%)	33 (12%)
2003	403	71 (9)	173 (43%)	40 (10%)	344 (85%)	51 (13%)	7 (0%)	1 (0%)	95 (24%)	96 (24%)	101 (25%)	64 (16%)	47 (12%)
2004	545	70 (9)	216 (40%)	60 (11%)	458 (84%)	78 (14%)	7 (0%)	2 (0%)	132 (24%)	116 (21%)	146 (27%)	102 (19%)	48 (9%)
2005	605	71 (9)	255 (42%)	60 (10%)	509 (84%)	83 (14%)	11 (0%)	2 (0%)	135 (22%)	129 (21%)	140 (23%)	137 (23%)	62 (10%)
2006	735	71 (9)	315 (43%)	75 (10%)	584 (79%)	135 (18%)	13 (0%)	3 (0%)	157 (21%)	168 (23%)	187 (25%)	142 (19%)	81 (11%)
2007	912	70 (9)	371 (41%)	83 (9%)	742 (81%)	141 (15%)	28 (0%)	1 (0%)	201 (22%)	207 (23%)	204 (22%)	190 (21%)	110 (12%)
2008	981	70 (9)	432 (44%)	73 (7%)	775 (79%)	177 (18%)	28 (0%)	1 (0%)	229 (23%)	243 (25%)	204 (21%)	188 (19%)	115 (12%)

2009	1045	69 (9)	463 (44%)	71 (7%)	773 (74%)	234 (22%)	29 (0%)	9 (0%)	229 (22%)	282 (27%)	247 (24%)	190 (18%)	96 (9%)
2010	1009	70 (9)	440 (44%)	72 (7%)	744 (74%)	209 (21%)	48 (0%)	8 (0%)	253 (25%)	249 (25%)	230 (23%)	176 (17%)	101 (10%)
2011	1024	69 (9)	461 (45%)	78 (8%)	715 (70%)	249 (24%)	54 (1%)	6 (0%)	229 (22%)	236 (23%)	250 (24%)	200 (20%)	109 (11%)
2012	1018	69 (9)	444 (44%)	69 (7%)	690 (68%)	273 (27%)	48 (0%)	7 (0%)	236 (23%)	269 (26%)	217 (21%)	182 (18%)	113 (11%)
2013	967	70 (9)	416 (43%)	65 (7%)	633 (65%)	272 (28%)	51 (0%)	11 (0%)	216 (22%)	251 (26%)	212 (22%)	184 (19%)	103 (11%)
2014	240	69 (9)	107 (45%)	17 (7%)	153 (64%)	66 (28%)	20 (0%)	1 (0%)	52 (22%)	54 (22%)	76 (32%)	32 (13%)	26 (11%)

Primary total hip replacement

Year of surgery	n	Age (mean (sd))	Gender: Male (n (%))	Rheumatoid arthritis (n (%))	RCS Charlson score (n (%)) 0	1	2	3+	IMD quintiles (n (%)) 1	2	3	4	5
1997	78	69 (10)	32 (41%)	5 (6%)	73 (94%)	5 (6%)	0 (0%)	0 (0%)	21 (27%)	15 (19%)	20 (26%)	16 (21%)	6 (8%)
1998	128	69 (10)	42 (33%)	8 (6%)	120 (94%)	7 (5%)	1 (0%)	0 (0%)	27 (21%)	26 (20%)	34 (27%)	23 (18%)	18 (14%)
1999	195	69 (12)	80 (41%)	11 (6%)	172 (88%)	22 (11%)	1 (0%)	0 (0%)	46 (24%)	47 (24%)	47 (24%)	32 (16%)	23 (12%)
2000	242	70 (10)	104 (43%)	6 (2%)	217 (90%)	22 (9%)	3 (0%)	0 (0%)	57 (24%)	79 (33%)	50 (21%)	37 (15%)	19 (8%)
2001	317	68 (10)	137 (43%)	14 (4%)	284 (90%)	28 (9%)	3 (0%)	2 (0%)	81 (26%)	87 (27%)	65 (21%)	54 (17%)	30 (9%)
2002	446	69 (10)	163 (37%)	27 (6%)	396 (89%)	44 (10%)	6 (0%)	0 (0%)	104 (23%)	115 (26%)	105 (24%)	75 (17%)	47 (11%)
2003	563	69 (10)	244 (43%)	35 (6%)	490 (87%)	66 (12%)	7 (0%)	0 (0%)	153 (27%)	140 (25%)	130 (23%)	99 (18%)	41 (7%)
2004	648	70 (10)	268 (41%)	36 (6%)	546 (84%)	89 (14%)	12 (0%)	1 (0%)	181 (28%)	166 (26%)	142 (22%)	100 (15%)	59 (9%)
2005	760	68 (11)	312 (41%)	51 (7%)	626 (82%)	114 (15%)	18 (0%)	2 (0%)	178 (23%)	190 (25%)	162 (21%)	153 (20%)	75 (10%)
2006	809	69 (11)	318 (39%)	48 (6%)	656 (81%)	131 (16%)	20 (0%)	2 (0%)	199 (25%)	210 (26%)	166 (21%)	150 (19%)	83 (10%)
2007	922	69 (11)	393 (43%)	56 (6%)	743 (81%)	154 (17%)	24 (0%)	1 (0%)	247 (27%)	240 (26%)	198 (21%)	167 (18%)	70 (8%)
2008	942	69 (11)	403 (43%)	52 (6%)	750 (80%)	158 (17%)	28 (0%)	6 (0%)	226 (24%)	227 (24%)	212 (23%)	181 (19%)	96 (10%)
2009	963	69 (11)	410 (43%)	46 (5%)	766 (80%)	166 (17%)	25 (0%)	6 (0%)	248 (26%)	259 (27%)	216 (22%)	166 (17%)	72 (7%)

2010	943	69 (11)	412 (44%)	57 (6%)	688 (73%)	222 (24%)	26 (0%)	7 (0%)	219 (23%)	250 (27%)	221 (23%)	154 (16%)	99 (10%)
2011	954	69 (10)	376 (39%)	64 (7%)	710 (74%)	191 (20%)	42 (0%)	11 (0%)	238 (25%)	256 (27%)	225 (24%)	170 (18%)	65 (7%)
2012	963	69 (10)	369 (38%)	66 (7%)	679 (71%)	224 (23%)	47 (0%)	13 (0%)	249 (26%)	226 (23%)	238 (25%)	166 (17%)	84 (9%)
2013	879	68 (11)	370 (42%)	43 (5%)	614 (70%)	213 (24%)	40 (0%)	12 (0%)	245 (28%)	215 (24%)	199 (23%)	145 (17%)	74 (8%)
2014	209	70 (10)	92 (44%)	14 (7%)	147 (70%)	52 (25%)	8 (0%)	2 (0%)	60 (29%)	46 (22%)	49 (23%)	35 (17%)	19 (9%)

Revision total knee replacement

					RCS Charlson score (n (%))				IMD quintiles (n (%))				
Year of surgery	n	Age (mean (sd))	Gender: Male (n (%))	Rheumatoid arthritis (n (%))	0	1	2	3+	1	2	3	4	5
1997	2	74 (1)	1 (50%)	0 (0%)	2 (100%)	0 (0%)	0 (0%)	0 (0%)	1 (50%)	1 (50%)	0 (0%)	0 (0%)	0 (0%)
1998	3	78 (2)	1 (33%)	2 (67%)	3 (100%)	0 (0%)	0 (0%)	0 (0%)	2 (67%)	0 (0%)	0 (0%)	1 (33%)	0 (0%)
1999	1	64 (-)	0 (0%)	1 (100%)	1 (100%)	0 (0%)	0 (0%)	0 (0%)	0 (0%)	0 (0%)	0 (0%)	1 (100%)	0 (0%)
2000	3	60 (19)	1 (33%)	3 (100%)	3 (100%)	0 (0%)	0 (0%)	0 (0%)	0 (0%)	1 (33%)	1 (33%)	0 (0%)	1 (33%)
2001	4	68 (13)	3 (75%)	0 (0%)	3 (75%)	1 (25%)	0 (0%)	0 (0%)	0 (0%)	2 (50%)	0 (0%)	1 (25%)	1 (25%)
2002	9	74 (9)	6 (67%)	1 (11%)	9 (100%)	0 (0%)	0 (0%)	0 (0%)	1 (11%)	6 (67%)	1 (11%)	1 (11%)	0 (0%)
2003	20	71 (11)	12 (60%)	3 (15%)	16 (80%)	4 (20%)	0 (0%)	0 (0%)	5 (25%)	2 (10%)	7 (35%)	5 (25%)	1 (5%)
2004	23	67 (10)	13 (57%)	4 (17%)	17 (74%)	4 (17%)	2 (0%)	0 (0%)	2 (9%)	8 (35%)	8 (35%)	4 (17%)	1 (4%)
2005	23	69 (11)	10 (43%)	4 (17%)	20 (87%)	3 (13%)	0 (0%)	0 (0%)	8 (35%)	4 (17%)	2 (9%)	8 (35%)	1 (4%)
2006	35	68 (9)	15 (43%)	6 (17%)	26 (74%)	8 (23%)	1 (0%)	0 (0%)	11 (31%)	8 (23%)	8 (23%)	6 (17%)	2 (6%)
2007	41	68 (10)	22 (54%)	4 (10%)	31 (76%)	5 (12%)	4 (1%)	1 (0%)	9 (22%)	5 (12%)	10 (24%)	13 (32%)	4 (10%)
2008	57	68 (10)	28 (49%)	9 (16%)	39 (68%)	17 (30%)	1 (0%)	0 (0%)	12 (21%)	12 (21%)	13 (23%)	11 (19%)	9 (16%)
2009	60	72 (10)	24 (40%)	7 (12%)	49 (82%)	10 (17%)	1 (0%)	0 (0%)	15 (25%)	17 (28%)	11 (18%)	14 (23%)	3 (5%)

2010	59	69 (10)	26 (44%)	8 (14%)	36 (61%)	20 (34%)	2 (0%)	1 (0%)	15 (25%)	15 (25%)	15 (25%)	10 (17%)	4 (7%)
2011	58	70 (10)	26 (45%)	6 (10%)	38 (66%)	18 (31%)	2 (0%)	0 (0%)	14 (24%)	16 (28%)	16 (28%)	9 (16%)	3 (5%)
2012	55	69 (10)	26 (47%)	3 (5%)	36 (65%)	15 (27%)	4 (1%)	0 (0%)	19 (35%)	12 (22%)	10 (18%)	12 (22%)	2 (4%)
2013	33	71 (9)	14 (42%)	3 (9%)	20 (61%)	9 (27%)	4 (1%)	0 (0%)	7 (21%)	9 (27%)	10 (30%)	6 (18%)	1 (3%)
2014	18	69 (9)	5 (28%)	1 (6%)	12 (67%)	5 (28%)	0 (0%)	1 (0%)	7 (39%)	2 (11%)	4 (22%)	3 (17%)	2 (11%)

Revision total hip replacement

Year of surgery	n	Age (mean (sd))	Gender: Male (n (%))	Rheumatoid arthritis (n (%))	RCS Charlson score (n (%))				IMD quintiles (n (%))				
					0	1	2	3+	1	2	3	4	5
1997	2	74 (11)	1 (50%)	1 (50%)	2 (100%)	0 (0%)	0 (0%)	0 (0%)	2 (100%)	0 (0%)	0 (0%)	0 (0%)	0 (0%)
1998	6	72 (8)	2 (33%)	0 (0%)	6 (100%)	0 (0%)	0 (0%)	0 (0%)	4 (67%)	2 (33%)	0 (0%)	0 (0%)	0 (0%)
1999	8	70 (14)	3 (38%)	1 (12%)	8 (100%)	0 (0%)	0 (0%)	0 (0%)	3 (38%)	3 (38%)	1 (12%)	1 (12%)	0 (0%)
2000	14	72 (11)	9 (64%)	2 (14%)	11 (79%)	3 (21%)	0 (0%)	0 (0%)	5 (36%)	1 (7%)	5 (36%)	1 (7%)	2 (14%)
2001	14	69 (10)	5 (36%)	2 (14%)	14 (100%)	0 (0%)	0 (0%)	0 (0%)	3 (21%)	2 (14%)	3 (21%)	5 (36%)	1 (7%)
2002	8	73 (11)	4 (50%)	2 (25%)	7 (88%)	1 (12%)	0 (0%)	0 (0%)	4 (50%)	1 (12%)	2 (25%)	0 (0%)	1 (12%)
2003	22	68 (11)	8 (36%)	7 (32%)	21 (95%)	1 (5%)	0 (0%)	0 (0%)	9 (41%)	5 (23%)	4 (18%)	2 (9%)	2 (9%)
2004	35	70 (10)	15 (43%)	6 (17%)	29 (83%)	6 (17%)	0 (0%)	0 (0%)	16 (46%)	7 (20%)	5 (14%)	3 (9%)	4 (11%)
2005	41	73 (10)	19 (46%)	7 (17%)	32 (78%)	9 (22%)	0 (0%)	0 (0%)	10 (24%)	9 (22%)	8 (20%)	8 (20%)	6 (15%)
2006	33	66 (12)	17 (52%)	5 (15%)	26 (79%)	3 (9%)	4 (1%)	0 (0%)	12 (36%)	8 (24%)	9 (27%)	1 (3%)	3 (9%)
2007	54	70 (10)	22 (41%)	5 (9%)	43 (80%)	10 (19%)	1 (0%)	0 (0%)	21 (39%)	16 (30%)	10 (19%)	3 (6%)	4 (7%)
2008	57	69 (11)	23 (40%)	8 (14%)	45 (79%)	10 (18%)	1 (0%)	1 (0%)	20 (35%)	12 (21%)	11 (19%)	10 (18%)	4 (7%)
2009	54	70 (13)	19 (35%)	9 (17%)	40 (74%)	12 (22%)	1 (0%)	1 (0%)	15 (28%)	18 (33%)	9 (17%)	9 (17%)	3 (6%)

2010	71	67 (12)	33 (46%)	7 (10%)	54 (76%)	14 (20%)	3 (0%)	0 (0%)	23 (32%)	20 (28%)	11 (15%)	9 (13%)	8 (11%)
2011	67	73 (11)	29 (43%)	4 (6%)	51 (76%)	7 (10%)	8 (1%)	1 (0%)	16 (24%)	14 (21%)	15 (22%)	12 (18%)	10 (15%)
2012	71	71 (9)	28 (39%)	14 (20%)	47 (66%)	15 (21%)	7 (1%)	2 (0%)	25 (35%)	15 (21%)	18 (25%)	10 (14%)	3 (4%)
2013	66	71 (9)	24 (36%)	10 (15%)	41 (62%)	17 (26%)	6 (1%)	2 (0%)	18 (27%)	20 (30%)	13 (20%)	8 (12%)	7 (11%)
2014	9	76 (14)	3 (33%)	1 (11%)	5 (56%)	3 (33%)	1 (0%)	0 (0%)	2 (22%)	2 (22%)	3 (33%)	1 (11%)	1 (11%)

LENGTH OF STAY BY YEAR OF SURGERY

Year	TKR		THR		TKR revision		THR revision	
	N	Mean (se)	N	Mean (se)	N	Mean (se)	N	Mean (se)
1997	45	18.89 (3.12)	78	14.28 (1.47)	2	12.5 (2.5)	3	27.33 (5.67)
1998	64	14.77 (0.78)	128	12.49 (0.42)	3	20 (2.08)	6	14.83 (1.08)
1999	114	12.79 (0.51)	195	12.1 (0.39)	1	18 (-)	8	18.75 (6.21)
2000	116	13.25 (1.09)	242	12.54 (0.64)	3	74 (59.03)	14	15 (1.79)
2001	170	12.13 (0.48)	319	11.97 (0.5)	4	15.25 (6.65)	14	21.86 (5.16)
2002	268	11.12 (0.42)	447	10.89 (0.26)	9	24 (7.11)	8	32.12 (12.92)
2003	403	10.32 (0.22)	561	10.3 (0.25)	21	21.29 (4.61)	23	22.48 (5.47)
2004	545	9.58 (0.3)	647	9.82 (0.29)	24	16.54 (3.61)	35	20.49 (3.42)
2005	604	9.11 (0.38)	761	9.33 (0.24)	22	19.64 (6.52)	40	21.48 (4.87)
2006	735	8.75 (0.27)	812	8.79 (0.22)	36	15.08 (3.35)	36	14.11 (2.87)
2007	913	8.1 (0.2)	923	8.25 (0.26)	41	13.41 (3.01)	58	15.28 (2.28)
2008	982	7.49 (0.24)	940	7.94 (0.3)	56	17.45 (4.1)	53	30.66 (5.38)
2009	1043	7.14 (0.21)	964	7.09 (0.18)	60	15.22 (2.74)	53	22.28 (4.33)
2010	1009	6.43 (0.11)	941	7.02 (0.17)	61	18.57 (5.55)	70	14.94 (3.05)
2011	1024	6.12 (0.1)	954	6.27 (0.13)	57	9.86 (1.35)	66	17.12 (2.55)
2012	1018	6.03 (0.16)	963	6.66 (0.3)	53	11.19 (2.03)	74	17.47 (3.05)
2013	967	5.89 (0.12)	877	5.83 (0.14)	35	15.4 (3.51)	63	11.87 (1.31)

2014	240	5.13 (0.15)	209	5.6 (0.3)	17	6.06 (0.8)	9	15.33 (4.78)

Number of study participants (N) with mean length of stay with standard error (se) by year. THR: total hip replacement, TKR: total knee replacement

HOSPITAL COSTS BY YEAR OF SURGERY

Year	Primary TKR		Primary THR		Revision TKR		Revision THR	
	N	Mean (se)	N	Mean (se)	N	Mean (se)	N	Mean (se)
1997	45	8516.45 (708.81)	78	7428.29 (359.68)	2	7562 (-)	3	9500.67 (2200.33)
1998	64	7590.65 (180.75)	128	7101.09 (98.33)	3	9677 (-)	6	7610.83 (362.58)
1999	114	7140.98 (128.49)	195	6921.47 (73.94)	1	8267 (-)	8	7244 (989.48)
2000	116	7168.41 (241.34)	242	6968.52 (103.49)	3	7562 (575.63)	14	7540.93 (500.08)
2001	170	6977.79 (100.8)	319	6839.25 (94.39)	4	6239.75 (1392.38)	14	8086.08 (670.15)
2002	268	6774.28 (89.49)	447	6669.12 (53.2)	9	9339.75 (1769.17)	8	10039.57 (2172.19)
2003	403	6590.4 (45.92)	561	6564.01 (54.2)	21	8544.84 (990.89)	23	8855 (907.28)
2004	545	6477.54 (68.11)	647	6417.76 (44.49)	24	7653.09 (493.94)	35	8442.61 (579.42)
2005	604	6393.04 (68.77)	761	6379.89 (53.06)	22	8306.64 (1423.54)	40	8688.02 (830.29)
2006	735	6401.5 (60.82)	812	6263 (40.8)	36	8026.77 (680.29)	36	7786.17 (468.81)
2007	913	6289.42 (42.25)	923	6155.92 (39.35)	41	8259.78 (595.51)	58	7942.64 (407.56)

2008	982	6194.13 (42.4)	940	6152.11 (58.67)	56	8249.2 (698.63)	53	10707.98 (989.17)
2009	1043	6202.38 (44.82)	964	5968.21 (24.92)	60	8056.98 (573.84)	53	8424.12 (614.65)
2010	1009	6107.99 (20.9)	941	6013.59 (31.66)	61	7829.78 (433.18)	70	8125.48 (608.8)
2011	1024	6108.19 (20.4)	954	5920.3 (24.32)	57	7130.88 (163.09)	66	8015.88 (383.55)
2012	1018	6136.84 (30.12)	963	6016.33 (47.13)	53	6906.06 (219.48)	74	8773.54 (596.93)
2013	967	6131.57 (22.07)	877	5900.26 (23.51)	35	8177.89 (737.29)	63	7523.76 (213.64)
2014	240	6053.24 (32.19)	209	5870.64 (50.52)	17	6761.94 (81.42)	9	7071.88 (615.47)

Number of study participants (N) with mean cost with standard error (se) by year. THR: total hip replacement, TKR: total knee replacement

PARTIAL EFFECT OF AGE ON LENGTH OF STAY AND COSTS

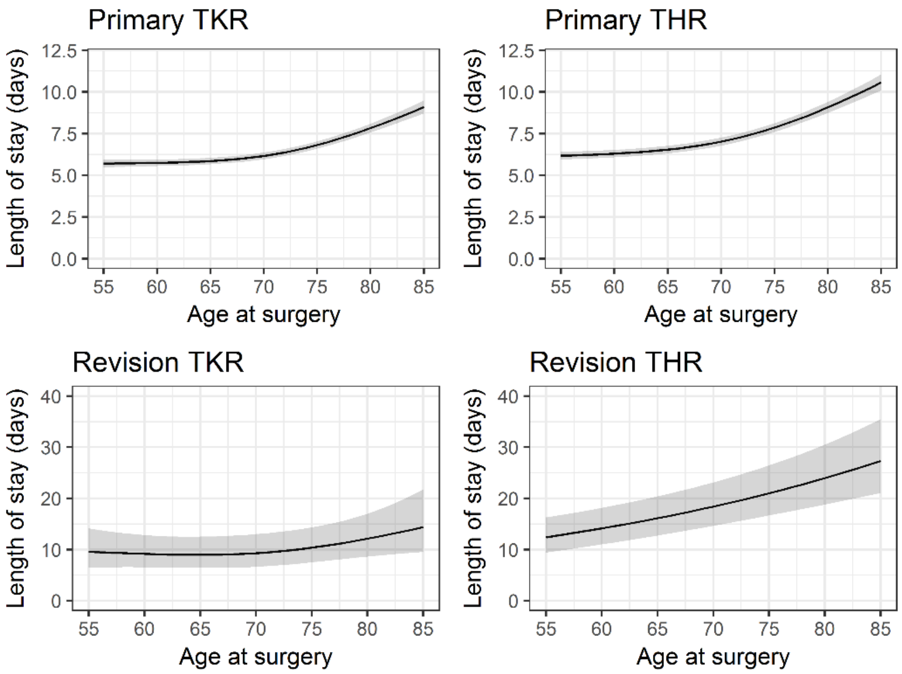

Partial effect of age at surgery on length of stay from multivariable regressions. Predicted length of stay by age while other explanatory variables are held fixed at their average (median if continuous and mode if categorical). TKR: total knee replacement, THR: total hip replacement.

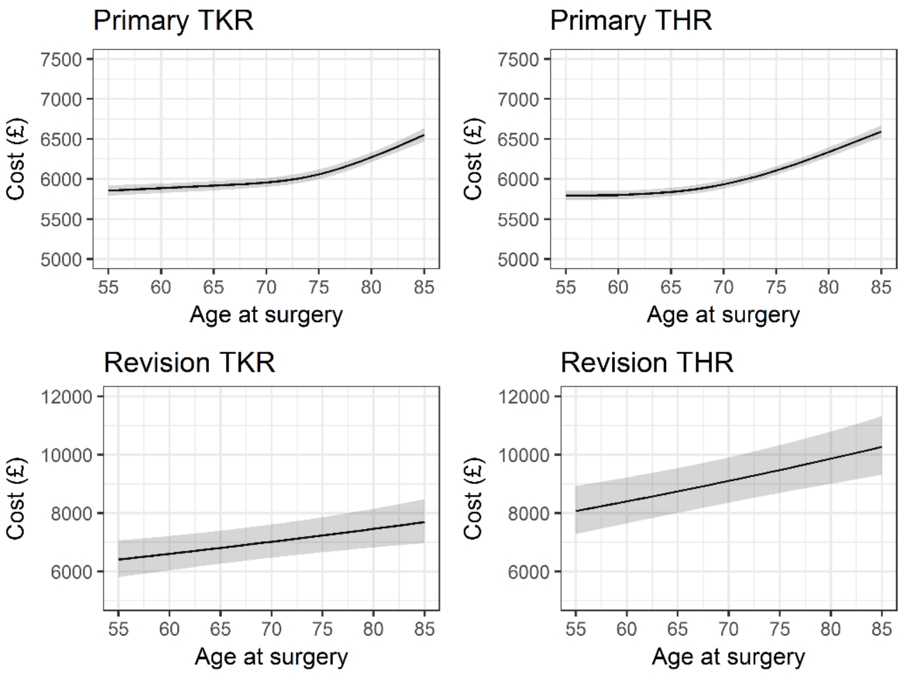

Partial effect of age at surgery on hospital costs (in 2017 GBP) from multivariable regressions. Predicted cost by age while other explanatory variables are held fixed at their average (median if continuous and mode if categorical). TKR: total knee replacement, THR: total hip replacement.
