# Supplementary material for: Trends and determinants of length of stay and hospital reimbursement following knee and hip replacement: evidence from linked primary care and NHS hospital records from 1997 to 2014
Source: BMJ Open. 2018 Jan 27;8(1):e019146. doi: 10.1136/bmjopen-2017-019146 (PMC5829869; doi:10.1136/bmjopen-2017-019146)
Supplement: Author's manuscript [file bmjopen-2017-019146.draft_revisions.pdf]

BMJ Open

BMJ Open is committed to open peer review. As part of this commitment we make the peer review history of every article we publish publicly available.

When an article is published we post the peer reviewers' comments and the authors' responses online. We also post the versions of the paper that were used during peer review. These are the versions that the peer review comments apply to.

The versions of the paper that follow are the versions that were submitted during the peer review process. They are not the versions of record or the final published versions. They should not be cited or distributed as the published version of this manuscript.

BMJ Open is an open access journal and the full, final, typeset and author-corrected version of record of the manuscript is available on our site with no access controls, subscription charges or pay-per-view fees (http://bmjopen.bmj.com).

If you have any questions on BMJ Open's open peer review process please email editorial.bmjopen@bmj.com

Trends and determinants of length of stay and hospital costs following knee and hip replacement: evidence from linked primary care and NHS hospital records from 1997 to 2014

Journal:	*BMJ Open*
Manuscript ID	bmjopen-2017-019146
Article Type:	Research
Date Submitted by the Author:	13-Aug-2017

Complete List of Authors:	Burn, Edward; University of Oxford Nuffield Department of Orthopaedics Rheumatology and Musculoskeletal Sciences<br>Edwards, Christopher; NIHR Wellcome Trust Clinical Research Facility, University Hospital Southampton<br>Murray, David; University of Oxford, Nuffield Department of Orthopaedics, Rheumatology & Musculoskeletal Sciences<br>Silman, Alan; University of Oxford Nuffield Department of Orthopaedics Rheumatology and Musculoskeletal Sciences<br>Cooper, Cyrus; University of Southampton and Southampton University Hospitals NHS Trust, MRC Lifecourse Epidemiology Unit; University of Oxford Nuffield Department of Orthopaedics Rheumatology and Musculoskeletal Sciences<br>Arden, Nigel; Nuffield Department of Orthopaedics; MRC Lifecourse Epidemiology Unit, Southampton University<br>Pinedo-Villanueva, Rafael; University of Oxford Nuffield Department of Orthopaedics Rheumatology and Musculoskeletal Sciences, Nuffield Department of Orthopaedics Rheumatology and Musculoskeletal Sciences; MRC Lifecourse Epidemiology Unit, Southampton University<br>Prieto-Alhambra, Daniel; University of Oxford Nuffield Department of Orthopaedics Rheumatology and Musculoskeletal Sciences; GREMPAL Research Group, Idiap Jordi Gol and CIBERFes, Universitat Autonoma de Barcelona and Instituto de Salud Carlos III

Keywords:	Joint Replacement, Knee Arthroplasty, Hip Arthroplasty, Length of Stay, Costs

SCHOLARONE™
Manuscripts

Trends and determinants of length of stay and hospital costs following knee and hip replacement: evidence from linked primary care and NHS hospital records from 1997 to 2014

Authors:

Edward Burn, MSc[1], Christopher J Edwards, FRCP,[2] David W Murray, MD,[1] Alan Silman, FMedSci,[1] Cyrus Cooper, FMedSci,[1,3] Nigel K Arden, FRCP,[1,3] Rafael Pinedo-Villanueva, PhD,[1,3] Daniel Prieto-Alhambra, PhD[1,4]

Affiliations:

[1]Nuffield Department of Orthopaedics, Rheumatology and Musculoskeletal Sciences, University of Oxford

2 NIHR Wellcome Trust Clinical Research Facility, University Hospital Southampton, Southampton, UK

3 MRC Lifecourse Epidemiology Unit, Southampton University, Southampton, UK

4 GREMPAL Research Group, Idiap Jordi Gol and CIBERFes, Universitat Autonoma de Barcelona and Instituto de Salud Carlos III, Barcelona, Spain

Correspondence to: Rafael Pinedo-Villanueva (rafael.pinedo@ndorms.ox.ac.uk)

Key words: Joint Replacement, Knee Arthroplasty, Hip Arthroplasty, Length of Stay, Costs

For peer review only

ABSTRACT

Objectives

To assess whether length of stay and hospital costs following total knee and hip replacement (TKR and THR) decreased between 1997 and 2014, the degree to which reductions can be explained by improved efficiency or changes in patient profiles, and the impact of individuals' characteristics on length of stay and costs.

Design

Cross-sectional study using routinely collected data.

Setting

NHS primary care records from 1995 to 2014 in the Clinical Practice Research Datalink (CPRD) were linked to hospital inpatient data from 1995 to 2014 in Hospital Episode Statistics (HES).

Participants

Study participants had a diagnosis of osteoarthritis (OA) or rheumatoid arthritis (RA).

Interventions

Primary TKR, primary THR, revision TKR, and revision THR.

Primary outcome measures

Length of stay and hospital costs.

Results

21,128 individuals with OA or RA were included, with 10,260 undergoing primary TKR, 10,961 THR, 505 TKR revision, and 633 THR revision. Length of stay fell significantly for primary TKR, primary THR and revision TKR, while costs fell significantly for primary TKR and primary THR between 1997 and 2014. The estimated effect of the year surgery took place remained similar when patient characteristics were included in multivariable regressions. Older age at surgery was associated with significantly increased length of stay and costs for both primary and revision procedures. A diagnosis of RA, other comorbidities and lower socioeconomic status were associated with significantly higher length of stay and costs for primary procedures. Male gender was associated with significantly shorter stays for primary procedures.

For peer review only

Conclusions

Average length of stay and hospital costs for joint replacement fell substantially from 1997 to 2014. While patient characteristics affect costs and length of stay, patient profiles have remained broadly stable over time. The observed reductions appear to be mostly explained by improved efficiency, suggesting that joint replacement is increasingly cost-effective.

ARTICLE SUMMARY

Strengths and limitations of this study

- Use of routinely collected data which provided real-world evidence of trends in length of stay and costs following primary knee and hip replacement and revision procedures.
- Patient characteristics were controlled for to assess whether trends in length of stay and costs were explained by changes in patient characteristics or improved efficiency.
- Codes used to identify diagnoses of osteoarthritis have not been fully evaluated.

Word count: 3,236

For peer review only

INTRODUCTION

Knee and hip replacements relieve pain and improve function for individuals with end-stage arthritis, leading to substantial gains in an individual's quality of life.[1,2] The procedures are a cost-effective use of healthcare resources, with their costs justified by the expected health gains for the individuals receiving them.[1,2] Rates of joint replacement grew substantially between 1991 and 2006 in the United Kingdom.[3] Demand is projected to continue to rise in the United States up to 2030 and, in turn, the number of revision procedures is also expected to increase.[4]

Satisfying rising demand will require either additional resources, which is unlikely given current funding constraints, or reducing the cost of the procedures. Length of stay is a key component of the overall cost of joint replacement. Greater efficiency should reduce the expected length of stay and hospital costs for an individual requiring surgery. If patient health outcomes are not compromised in the process, greater efficiency would make joint replacements a more cost-effective procedure.

Length of stay following joint replacement has fallen substantially.[5][6][7][8][9][10][11][12] Although such reductions may be explained by improved efficiency, they may also be explained by changes in patient profiles over time. As patient characteristics are likely to influence length of stay, changes in these characteristics would partially explain the observed changes in stay length. Individuals at greater risk of poor health outcomes, due to either pre-operative comorbidities or post-operative complications, can be expected to have longer stays. Social determinants can also be expected to influence length of stay. Understanding the influence of such factors could inform targeted interventions for those at highest risk of a prolonged hospital stay.

We assessed the extent to which length of stay following joint replacement decreased in the English National Health Service from 1997 to 2014 and whether such changes have been translated into lower hospital costs. We considered the degree to which observed changes can be explained by improved efficiency or changes in patient profiles, and the impact of individuals' characteristics on length of stay and hospital costs.

For peer review only

METHODS

Study Design

Individuals with a diagnosis of osteoarthritis (OA) or rheumatoid arthritis (RA) were identified from primary care records. Linked hospital records were used to identify the procedures of interest following diagnosis: primary TKR, primary total hip replacement (THR), revision TKR, and revision THR. Length of stay and hospital costs for these surgeries were calculated. Trends in length of stay and hospital costs over time were assessed using univariable generalised linear models. Patient characteristics were added as explanatory variables to the models to consider whether trends were explained by changes in efficiency or changes in patient profiles.

Setting

Primary care records were derived from the Clinical Practice Research Datalink (CPRD). CPRD contains medical records and demographic details for around 7% of the UK population, with those included representative of the general population.[13] A subset of practices within CPRD were linked to inpatient hospital records, provided by Hospital Episode Statistics Admitted Patient Care (HES APC). HES APC contains data on all NHS hospital admissions in England, with each uninterrupted inpatient stay at one hospital recorded as a spell (the time a patient spent in a particular hospital).[14] The CPRD extract used to identify diagnoses began in 1995. The HES APC extract used to identify events of interest began in 1997. Linked data from both databases were extracted up to 2014.

Participants

We extracted CPRD records for individuals with a diagnosis of either OA or RA. Study participants were identified separately for procedures relating to the knee and hip. For procedures relating to the knee, CPRD records were extracted for those individuals with a diagnosis recorded for RA or knee OA. For procedures relating to the hip, records were extracted for individuals with a diagnosis of RA or hip OA.

Some individuals had diagnostic codes for both RA and OA. It was not possible to determine which of their diagnoses drove the decision for joint replacement. The need a joint replacement for those with RA is often dependent on the development of secondary OA. Diagnosis of RA was therefore taken as the index diagnosis from which follow-up started when patients had both diagnoses recorded.

For peer review only

CPRD records were then linked with HES APC data and procedures of interest after the index diagnosis were identified. Only the first instance of each procedure for an individual was included in the analysis, and any bilateral procedures were excluded. Although no individuals contributed multiple records of the same surgery, an individual could provide data on multiple procedures.

Variables

Diagnoses of RA, knee OA, and hip OA were identified using clinical (READ) codes within CPRD. Events of interest (TKR, TKR revision, THR, and THR revision) were identified using intervention (OPCS) codes in HES APC.

The date of a procedure recorded in HES APC was used to identify the year of surgery and, given an individual's year of birth recorded in CPRD, age at surgery. Gender was recorded in CPRD. Diagnosis codes in HES APC were used to identify diseases included in the Royal College of Surgeons (RCS) Charlson score, a measure of comorbidities, and calculate the overall summary score (0, 1, 2 or 3+).[15] Index of Multiple Deprivation (IMD) quintiles, a measure of socio-economic status (with IMD quintile 5 implying the highest level of deprivation), were derived from CPRD.

Length of stay was recorded within HES APC by days for the spell in which a procedure occurred. Hospital spells were assigned to Healthcare Resource Groups (HRGs), which group clinically similar treatments using common levels of healthcare resources. The 2017/18 draft prices were used to estimate hospital costs of spells based on the HRG, the method of admission (elective or non-elective), the expected physiotherapy cost, and any stay longer than the trim-point, which is the upper limit of length of stay incorporated in the HRG cost. While variation between individuals' costs were influenced by HRG codes and type of admission, high costs were driven primarily by stays longer than the trim-point. The estimated costs reflect current reimbursement to hospitals from the NHS, not the accounting costs incurred by hospitals or historical reimbursement rate. Secondary diagnosis codes that were ineligible for grouping were dropped from the record. The cost was classified as missing if the primary diagnosis or any other variable required for the grouper software was ineligible.

For peer review only

Statistical methods

Trends in length of stay and hospital costs over time for each procedure were assessed using univariable regression models that used the year of surgery as the explanatory factor. Multivariable regressions that included patient characteristics were also specified. We compared the estimated effect of year of surgery on length of stay and hospital costs with and without patient characteristics to determine the extent to which a trend over time could be explained by change in patient characteristics or improvements in efficiency. A similar approach has previously been used to assess whether variations in length of stay between types of providers are due to differences in efficiency or patient selection.[16]

As both length of stay and hospital costs are non-negative and right-skewed, generalised linear models (GLMs) with a gamma distribution and a log-link were specified.[17] For continuous variables, non-linearity was incorporated using restricted cubic splines. The Bayesian information criterion (BIC) was used to decide whether to specify a variable's relationship as non-linear and, if included, how many knots to include. Compared to the Akaike information criterion (AIC), BIC favours parsimony and has been recommended for explanatory models.[18]

Year of surgery, diagnosis (RA or OA), age, gender, RCS Charlson score, and IMD quintile were included as explanatory variables in each of the multivariable models. The code for RA was omitted from calculations of the RCS Charlson score as RA was specified as a separate variable. The two highest RCS Charlson score categories, 2 and 3+, were combined into the category 2+ as there were few scores of 3 or more.

The only explanatory variable with missing data was the IMD quintile, which was missing for 9 TKR and 7 THR; these individuals were omitted from all regressions. There were no missing data for length of stay. Costs could not be calculated for the 2.3% of spells that the HRG grouper did not produce a valid HRG code for. Spells without HRG codes were dropped from the regressions on hospital costs, but were included in the analysis of length of stay.

Data analysis was primarily undertaken in R 3.3.1,[19] using dplyr[20] for data manipulation, rms[21] for fitting regression models, and ggplot2[22] to produce plots. HRG4+ codes were derived using the 2015/16 Reference Costs Grouper.[23]

For peer review only

RESULTS

Study participants

Records from 21,128 patients with OA or RA were included in the analysis, with 10,260 undergoing primary TKR, 10,961 primary THR, 505 TKR revision, and 633 THR revision in the study period. Inclusion of study participants is described in a flow chart in Figure 1. Around 2% of individuals had diagnoses of both OA and RA. RA was taken as the index diagnosis for these individuals.

Individuals' characteristics at the time of surgery are described in Table 1 and are stratified by year of surgery in the appendix. Age, gender, and socio-economic status, measured by IMD Quintile, remained relatively stable over the study period. While the proportion of patients with a diagnosis of RA fell over time, the number of comorbidities at surgery, as measured by the RCS Charlson score, increased.

Trends in expected length of stay and hospital costs over time

Observed length of stay and hospital costs by year of surgery are detailed in the appendix. The estimated effects of year of surgery on length of stay from univariable regressions are presented in Figure 2. Expected length of stay fell from 16.0 days (95% confidence interval: 14.9 to 17.2) in 1997 to 5.4 (5.2 to 5.6) in 2014 for primary TKR, from 14.4 (13.7 to 15.0) to 5.6 (5.4 to 5.8) for primary THR, from 29.8 (17.5 to 50.5) to 11.0 (8.3 to 14.6) for revision TKR, and from 18.3 (11.6 to 28.9) to 12.5 (9.3 to 16.8) for revision THR. Including patient characteristics in the regression models had little effect on the association between year of surgery and length of stay and hospital costs, as shown in Table 2. For example, multivariable adjustment for patient characteristics changed the expected reduction in length of stay for primary THR from 5% (RE: 0.95 [95% CI: 0.94 to 0.95]) to 6% per year (RE: 0.94 [95% CI: 0.94 to 0.95]).

Estimated trends in hospital costs from univariable regressions were broadly similar to those estimated for length of stay (Figure 3). Expected mean hospital costs fell from £7,634 (£7,464 to £7,808) in 1997 to £6,097 (£6,016 to £6,179) in 2014 for primary TKR, from £7,300 (£7,187 to £7,414) to £5,888 (£5,827 to £5,949) for primary THR, from £9,088 (£7,727 to £10,689) to £7,296 (£6,717 to £7,926) for revision TKR, and from £7,655 (£6,385 to £9,177) to £7,564 (£6,743 to £8,485) for revision THR. Including patient characteristics in the regression models did not remove the estimated association between year of surgery

For peer review only

and hospital costs, as shown in Table 3. For example, the expected reduction in length of stay for revision TKR was 1% per year (RE: 0. 99 [95% CI: 0.97 to 1.00]) in the unadjusted model and 1% per year (RE: 0. 99 [95% CI: 0.98 to 1.00]) in the multivariable model.

Impact of patient characteristics on expected length of stay and hospital costs

Tables 2 and 3 detail the relative effects of patient characteristics on length of stay and hospital costs from the fully specified models. Higher age at surgery was associated with longer stays for each procedure. Where the effect of age was non-linear, its impact was more pronounced among older patients. Holding all other explanatory variables at their average, expected length of stay increased from 5.7 (5.5 to 5.9) for a 55-year-old to 9.1 (8.7 to 9.5) for an 85-year-old undergoing primary TKR, from 6.2 (5.9 to 6.4) to 10.6 (10.1 to 11.0) for primary THR, from 9.6 (6.5 to 14.1) to 14.4 (9.5 to 21.8) for revision TKR, and from 12.4 (9.4 to 16.3) to 27.3 (21.0 to 35.5) for revision THR. Similar upward trends were estimated for hospital costs for each of the procedures of interest. With other explanatory variables held at their average value, expected cost increased from £5,856 (£5,791 to £5,923) for a 55-year-old to £6,553 (£6,471 to £6,636) for an 85-year-old undergoing primary TKR, from £5,792 (£5,734 to £5,850) to £6,596 (£6,520 to £6,672) for primary THR, from £6,409 (£5,811 to £7,069) to £7,691 (£6,976 to £8,480) for revision TKR, and from £8,071 (£7,292 to £8,934) to £10,269 (£9,315 to £11,320) for revision THR. Plots of the partial effect of age on length of stay and hospital costs are provided in the appendix.

DISCUSSION

Principal findings

Length of stay for primary TKR and THR fell significantly in the English NHS between 1997 and 2014. Expected length of stay fell by 11 days for primary TKR and 9 days for primary THR. The trajectory of the fall in expected length of stay was estimated as linear for primary THR, but had slowed somewhat towards the end of the study period for primary TKR. Length of stay also fell significantly for revision TKR, by 19 days between 1997 and 2014. Although a drop of 6 days was estimated for revision THR, this fall was not statistically significant. Hospital costs also fell significantly for primary procedures, with reductions of £1,537 for primary TKR and £1,412 for primary THR. The trajectories of these reductions in costs were broadly in line with those of length of stay. The estimated reductions in costs for the revision procedures, £1,792 for revision TKR and £91 for revision THR, were not significant.

For peer review only

Age at surgery was associated with increased length of stay and costs for each procedure with, in general, greater increases among older patients. RA and other comorbidities were associated with significantly higher length of stay and hospital costs. Lower socioeconomic status was associated with a significant increase in length of stay and costs for primary procedures. Male gender was associated with significantly shorter stays for primary procedures.

While the age, gender, and socioeconomic status of those receiving joint replacement and revision surgery remained stable over time, individuals undergoing surgery had more comorbidities in more recent years although the proportion diagnosed with RA fell. Controlling for patient characteristics had little effect on the association between year of surgery and length of stay or hospital costs. These results imply that the downward trends in length of stay and hospital costs are generally explained by increased efficiency.

Strength and limitations of this study

Routinely collected data is not collected primarily to inform research leading to a number of possible limitations. Coding accuracy is of particular concern. While the diagnosis of RA[24] and the coding of primary TKR and primary THR[25] have previously been validated in CPRD and HES APC respectively, OA coding has not been thoroughly evaluated. If those misclassified as not having an OA diagnosis differed systematically from those with a recorded OA diagnosis, the external validity of our results may have been affected.

A number of patient characteristics were controlled for to assess whether trends in length of stay and costs were explained by changes in patient characteristics or improved efficiency. These included age, gender, comorbidities and socioeconomic status. However, other patient characteristics may have changed over time and influenced length of stay or costs.

A further limitation of this study is that we only examined the length of stay and hospital costs of the spell for each individual's first recorded primary and revision procedure. This may have led to an underestimation of costs. In particular, revision procedures can have multiple stages and subsequent revision procedures may be required, which may lead to a higher overall cost from needing a revision than reported here.

The primary strength of this study is that it was informed by routinely collected data, providing real-world evidence of trends in length of stay and hospital costs. Sample sizes were large, particularly for primary TKR and primary THR, allowing a thorough analysis of

For peer review only

trends over time, and the data were representative of the experience of the NHS in England. The costs estimated here reflect reimbursement to hospitals by the NHS and so best reflect the relevant costs for overall decision-making.

Study findings in context

Previous studies have also found length of stay to have fallen over recent decades following TKR and THR.[6][7][8][9][10][11][12] In the US, however, the costs of joint replacement have been rising, with the falling length of stay only slowing this increase.[26][27] The downward trends in length of stay and hospital costs observed in this study appear to be primarily due to increased efficiency rather than changes in patient characteristics. Efficiency gains have likely been achieved through a combination of factors, with changes in the procedure itself and in provider policies. Unicompartmental arthroplasty in knee replacement and minimally invasive surgery are both associated with faster post-operative recovery.[28][29] Enhanced recovery programmes have become increasingly prominent in orthopaedic surgery, aiming to standardise routine perioperative care, reduce length of hospital stay, and promote rapid recovery.[30] Such programmes reduce length of stay[31] with either equivalent[30][32] or improved health outcomes[31]. The number of specialised public treatment centres and private treatment centres has also increased, with such centres associated with shorter stays.[16]

As observed here, the age and gender of those undergoing surgery has been relatively stable between 1991 and 2006.[3] Older age at surgery, particularly those over 80[6][33], and of lower income[33] are both associated with longer stays. While gender has typically not been found to have an effect on length of stay[6] or costs[34], being female was associated with longer stays following TKR in one study.[33]

Over the study period individuals appear to have an increasing number of comorbidities at the time of surgery for all of the procedures considered. This trend has previously been reported in the US.[7][8][27] Comorbidities have been associated with longer hospital stays and greater costs following primary joint replacement.[33][34] The proportion of those undergoing primary TKR with a diagnosis RA though fell over time in this study. This trend has also previously been reported and is likely explained by therapeutic advances, such as the introduction of biologic therapies.[35] The relative impact of RA on length of stay and hospital costs is not well known, but the increases estimated here may be explained by

For peer review only

greater morbidity before surgery and the use of medications such as steroids and immunosuppressants.

Policy implications and areas for future research

Expected length of stay and hospital costs associated with joint replacement fell significantly between 1997 and 2014. These reductions do not appear to have been driven by changes in patient profiles, but rather improved efficiency. If health outcomes have not worsened, joint replacement will have become increasingly cost-effective.

With stretched healthcare budgets, further improvements in efficiency will likely be necessary for supply to keep pace with the rising demand for joint replacement procedures. Improvements in efficiency can continue, for example through the use of outpatient knee replacement.[41] Additional research into the drivers of the efficiency gains identified in this study would help inform future policy decisions.

While the proportion of patients undergoing surgery with a prior diagnosis of RA has fallen, patients increasingly have other comorbidities at the time of these surgeries. With comorbidities associated with longer stays and greater hospital costs, targeted interventions to minimise prolonged hospital stays can be expected to grow in importance.

For peer review only

ACKNOWLEDGEMENTS

The authors would like to thank Miss Susan Thwaite (National Rheumatoid Arthritis Society) for her role as the patient and public representative and her role in the study steering committee. We also thank Dr Jennifer A. de Beyer of the Centre for Statistics in Medicine, University of Oxford, for English language editing.

FUNDING

DPA is funded by a National Institute for Health Research Clinician Scientist award (CS-2013-13-012). This article presents independent research funded by the National Institute for Health Research (NIHR). The views expressed are those of the authors and not necessarily those of the NHS, the NIHR or the Department of Health. This work was supported by the NIHR Biomedical Research Centre, Oxford.

CONTRIBUTORS

EB, CE, DWM, AS, CC, NKA, RPV and DPA all made substantial contributions to conception and design of the study. EB, RPV and DPA undertook the statistical analysis. EB, RPV and DPA drafted the manuscript with CE, DWM, AS, CC, and NKA revising it for important intellectual content. All authors read and approved the final manuscript.

COMPETING INTERESTS

All authors have completed the ICMJE uniform disclosure form at www.icmje.org/coi_disclosure.pdf and declare: NKA has received personal fees from Freshfields Bruckhaus Deringer, Bioventus, Flexion, Merck, and Regeneron, all outside the submitted work. DPA reports grants from Amgen, Servier and UCB Biopharma, and non-financial support from Amgen, all outside the submitted work. DWM reports grants and personal fees from Zimmer Biomet. In addition, DWM has various patents related to Unicompartmental Knee Replacement (Zimmer Biomet) with royalties paid.

ETHICAL APPROVAL

Not required.

DATA SHARING

CPRD data with HES linkage were provided under a licence that does not permit sharing. Data are obtainable from CPRD subject to a full application.

For peer review only

REFERENCES

1 Pivec R, Johnson AJ, Mears SC, *et al.* Hip arthroplasty. *Lancet* 2012;**380**:1768–77. doi:10.1016/S0140-6736(12)60607-2

2 Carr AJ, Robertsson O, Graves S, *et al.* Knee replacement. *Lancet* 2012;**379**:1331–40. doi:10.1016/s0140-6736(11)60752-6

3 Culliford DJ, Maskell J, Beard DJ, *et al.* Temporal trends in hip and knee replacement in the United Kingdom: 1991 TO 2006. *J Bone Jt Surg - Br Vol* 2010;**92**–**B**:130–5. doi:10.1302/0301-620X.92B1.22654

4 Kurtz S, Ong K, Lau E, *et al.* Projections of primary and revision hip and knee arthroplasty in the United States from 2005 to 2030. *J Bone Joint Surg Am* 2007;**89**:780–5. doi:10.2106/JBJS.F.00222

5 Cram P, Lu X, Kates SL, *et al.* Total Knee Arthroplasty Volume, Utilization, and Outcomes Among Medicare Beneficiaries, 1991-2010. *JAMA* 2012;**308**:1227. doi:10.1001/2012.jama.11153

6 Smith IDM, Elton R, Ballantyne JA, *et al.* Pre-operative predictors of the length of hospital stay in total knee replacement. *J Bone Jt Surg - Br Vol* 2008;**90**–**B**:1435–40. doi:10.1302/0301-620X.90B11.20687

7 Cram P, Lu X, Kates SL, *et al.* Total Knee Arthroplasty Volume, Utilization, and Outcomes Among Medicare Beneficiaries, 1991-2010. *JAMA* 2012;**308**:1227. doi:10.1001/2012.jama.11153

8 Cram P. Clinical Characteristics and Outcomes of Medicare Patients Undergoing Total Hip Arthroplasty, 1991-2008. *Jama* 2011;**305**:1560. doi:10.1001/jama.2011.478

9 Cookson R, Laudicella M. Do the poor cost much more? The relationship between small area income deprivation and length of stay for elective hip replacement in the English NHS from 2001 to 2008. *Soc Sci Med* 2011;**72**:173–84. doi:10.1016/j.socscimed.2010.11.001

10 Husted H, Jensen CM, Solgaard S, *et al.* Reduced length of stay following hip and knee arthroplasty in Denmark 2000-2009: From research to implementation. *Arch Orthop*

Trauma Surg 2012;**132**:101–4. doi:10.1007/s00402-011-1396-0

11 Jimenez García R, Villanueva Martínez M, Fernández de Las-Penas C, Hernandez Barrera V, Rios Luna A, Carrasco Garrido P, Lopez de Andres A, Jimenez Trujillo I, San Roman Montero J, Gil de Miguel A. Trends in primary total hip arthroplasty in Spain from 2001 to 2008: Evaluating changes in demographics, comorbidity, incidence rates, length of stay, costs and mortality. *BMC Musculoskelet Disord* 2011;**12**:43. doi:10.1186/1471-2474-12-43

12 Pamilo KJ, Peltola M, Mäkelä K, *et al.* Is hospital volume associated with length of stay, re-admissions and reoperations for total hip replacement? A population-based register analysis of 78 hospitals and 54,505 replacements. *Arch Orthop Trauma Surg* 2013;**133**:1747–55. doi:10.1007/s00402-013-1860-0

13 Herrett E, Gallagher AM, Bhaskaran K, *et al.* Data Resource Profile: Clinical Practice Research Datalink (CPRD). *Int J Epidemiol* 2015;**44**:827–36. doi:10.1093/ije/dyv098

14 Herbert A, Wijlaars L, Zylbersztejn A, *et al.* Data Resource Profile: Hospital Episode Statistics Admitted Patient Care (HES APC). *Int J Epidemiol* 2017;:1–10. doi:10.1093/ije/dyx015

15 Armitage JN, Van Der Meulen JH. Identifying co-morbidity in surgical patients using administrative data with the Royal College of Surgeons Charlson Score. *Br J Surg* 2010;**97**:772–81. doi:10.1002/bjs.6930

16 Siciliani L, Sivey P, Street A. DIFFERENCES IN LENGTH OF STAY FOR HIP REPLACEMENT BETWEEN PUBLIC HOSPITALS, SPECIALISED TREATMENT CENTRES AND PRIVATE PROVIDERS: SELECTION OR EFFICIENCY? *Health Econ* 2013;**22**:234–42. doi:10.1002/hec.1826

17 Mihaylova B, Briggs A, O'Hagan A, *et al.* Review of statistical methods for analysing healthcare resources and costs. *Health Econ* 2011;**20**:897–916. doi:10.1002/hec.1653

18 Aho K, Derryberry D, Peterson T. Model selection for ecologists: the worldview of AIC and BIC. *Ecology* 2014;**95**:631–6. doi:10.1890/13-1452.1

19 R Core Team. R: A language and environment for statistical computing. R Foundation

For peer review only

for Statistical Computing, Vienna, Austria. 2016.

20 Wickham H, Francois R. dplyr: A Grammar of Data Manipulation. R package version 0.5.0. 2016.

21 Harrell , FE. rms: Regression Modeling Strategies. R package version 5.1-0. 2017.

22 Wickham H. *ggplot2: Elegant Graphics for Data Analysis*. New York: : Springer-Verlag 2009. doi:10.1007/978-0-387-98141-3

23 NHS Digital. No Title.

24 Thomas SL, Edwards CJ, Smeeth L, *et al.* How accurate are diagnoses for rheumatoid arthritis and juvenile idiopathic arthritis in the general practice research database? *Arthritis Care Res* 2008;**59**:1314–21. doi:10.1002/art.24015

25 Hawley S, Delmestri A, Judge A, *et al.* Total Hip and Knee Replacement Among Incident Osteoarthritis and Rheumatoid Arthritis Patients Within the UK Clinical Practice Research Datalink (CPRD) Compared to Hospital Episode Statistics (HES): A Validation Study. *Pharmacoepidemiol Drug Saf* 2016.

26 Cary MP, Baernholdt M, Merwin EI. Changes in Payment Regulation and Acute Care Use for Total Hip Replacement: Trends in Length of Stay, Costs, and Discharge, 1997-2012. *Rehabil Nurs* 2016;**41**:67–77. doi:10.1002/rnj.210

27 Molloy IB, Martin BI, Moschetti WE, *et al.* Effects of the Length of Stay on the Cost of Total Knee and Total Hip Arthroplasty from 2002 to 2013. *J Bone Jt Surg* 2017;**99**:402–7. doi:10.2106/JBJS.16.00019

28 Röttger J, Scheller-Kreinsen D, Busse R. Patient-level hospital costs and length of stay after conventional versus minimally invasive total hip replacement: A propensity-matched analysis. *Value Heal* 2012;**15**:999–1004. doi:10.1016/j.jval.2012.06.008

29 Liddle AD, Judge A, Pandit H, *et al.* Adverse outcomes after total and unicompartmental knee replacement in 101 330 matched patients: a study of data from the National Joint Registry for England and Wales. *Lancet* 2014;**384**:1437–45. doi:10.1016/s0140-6736(14)60419-0

30 Maempel JF, Walmsley PJ. Enhanced recovery programmes can reduce length of stay

For peer review only

after total knee replacement without sacrificing functional outcome at one year. *Ann R Coll Surg Engl* 2015;**97**:563–7. doi:10.1308/rcsann.2015.0016

31 Khan SK, Malviya A, Muller SD, *et al.* Reduced short-term complications and mortality following Enhanced Recovery primary hip and knee arthroplasty: results from 6,000 consecutive procedures. *Acta Orthop* 2014;**85**:26–31. doi:10.3109/17453674.2013.874925

32 Jones EL, Wainwright TW, Foster JD, *et al.* A systematic review of patient reported outcomes and patient experience in enhanced recovery after orthopaedic surgery. *Ann R Coll Surg Engl* 2014;**96**:89–94. doi:10.1308/003588414X13824511649571

33 El Bitar YF, Illingworth KD, Scaife SL, *et al.* Hospital Length of Stay following Primary Total Knee Arthroplasty: Data from the Nationwide Inpatient Sample Database. *J Arthroplasty* 2015;**30**:1710–5. doi:10.1016/j.arth.2015.05.003

34 Shah AN, Vail TP, Taylor D, *et al.* Comorbid illness affects hospital costs related to hip arthroplasty: Quantification of health status and implications for fair reimbursement and surgeon comparisons. *J Arthroplasty* 2004;**19**:700–5. doi:10.1016/j.arth.2004.02.034

35 Harty L, O'Toole G, FitzGerald O. Profound reduction in hospital admissions and musculoskeletal surgical procedures for rheumatoid arthritis with concurrent changes in clinical practice (1995-2010). *Rheumatology (Oxford)* 2015;**54**:666–71. doi:10.1093/rheumatology/keu340

For peer review only

Table 1. Patient characteristics by procedure

	Primary TKR	Primary THR	Revision TKR	Revision THR
Number of study participants	10,260	505	10,961	633
Age (mean (sd))	70.01 (9.24)	69.43 (9.93)	68.93 (10.53)	70.34 (10.91)
Gender: male (n (%))	4426 (43.1)	233 (46.1)	4525 (41.3)	264 (41.7)
Year of surgery (median [IQR])	2009 [2006, 2011]	2009 [2007, 2011]	2008 [2005, 2011]	2009 [2006, 2011]
Diagnoses recorded in CPRD				
Rheumatoid arthritis (n (%))	851 (8.3)	65 (12.9)	639 (5.8)	92 (14.5)
Diagnoses recorded in HES APC				
Myocardial infarction (n (%))	138 (1.3)	6 (1.2)	140 (1.3)	14 (2.2)
Congestive cardiac failure (n (%))	96 (0.9)	8 (1.6)	137 (1.2)	14 (2.2)
Peripheral vascular disease (n (%))	101 (1.0)	10 (2.0)	102 (0.9)	8 (1.3)
Cerebrovascular disease (n (%))	54 (0.5)	3 (0.6)	56 (0.5)	5 (0.8)
Dementia (n (%))	25 (0.2)	0 (0.0)	37 (0.3)	10 (1.6)
Chronic pulmonary disease (n (%))	1091 (10.6)	58 (11.5)	998 (9.1)	55 (8.7)
Rheumatological disease (n (%))	593 (5.8)	38 (7.5)	474 (4.3)	50 (7.9)
Liver disease (n (%))	20 (0.2)	0 (0.0)	22 (0.2)	5 (0.8)
Diabetes mellitus (n (%))	991 (9.7)	56 (11.1)	755 (6.9)	45 (7.1)
Hemiplegia or paraplegia (n (%))	18 (0.2)	4 (0.8)	17 (0.2)	5 (0.8)
Renal disease (n (%))	202 (2.0)	11 (2.2)	235 (2.1)	20 (3.2)
Any malignancy (n (%))	78 (0.8)	7 (1.4)	97 (0.9)	9 (1.4)
Metastatic solid tumour (n (%))	6 (0.1)	1 (0.2)	10 (0.1)	2 (0.3)
AIDS HIV infection (n (%))	0 (0.0)	0 (0.0)	1 (0.0)	0 (0.0)
RCS Charlson score (n (%))				
0	7443 (72.5)	342 (67.7)	8413 (76.8)	455 (71.9)
1	2302 (22.4)	130 (25.7)	2110 (19.3)	124 (19.6)
2	437 (4.3)	29 (5.7)	353 (3.2)	45 (7.1)
3+	78 (0.8)	4 (0.8)	85 (0.8)	9 (1.4)
IMD quintiles (n (%))				

For peer review only

1	2330 (22.7)	128 (25.3)	2779 (25.4)	208 (32.9)
2	2487 (24.2)	120 (23.8)	2794 (25.5)	155 (24.5)
3	2383 (23.2)	117 (23.2)	2479 (22.6)	127 (20.1)
4	1930 (18.8)	105 (20.8)	1923 (17.5)	84 (13.3)
5	1121 (10.9)	35 (6.9)	980 (8.9)	59 (9.3)
Missing	9 (0.1)	0 (0.0)	6 (0.1)	0 (0.0)
Ethnicity (n (%))				
Non-White	240 (2.3)	15 (3.0)	60 (0.5)	6 (0.9)
White	9073 (88.4)	460 (91.1)	9643 (88.0)	575 (90.8)
Missing	947 (9.2)	30 (5.9)	1258 (11.5)	52 (8.2)

Table 2. Multivariable regressions for length of stay

	Primary TKR (RE (95% CI))		Primary THR (RE (95% CI))		Revision TKR (RE (95% CI))		Revision THR (RE (95% CI))	
	Unadjusted	Adjusted	Unadjusted	Adjusted	Unadjusted	Adjusted	Unadjusted	Adjusted
Year of surgery	0.93 (0.92 to 0.94)	0.93 (0.93 to 0.94)	0.95 (0.94 to 0.95)	0.94 (0.94 to 0.95)	0.94 (0.90 to 0.99)	0.96 (0.92 to 1.00)	1.02 (0.96 to 1.07)	1.02 (0.96 to 1.07)
Year of surgery†	1.01 (1.00 to 1.02)	1.01 (1.00 to 1.02)					0.93 (0.87 to 1.00)	0.91 (0.85 to 0.98)
Age		1.00 (1.00 to 1.00)		1.00 (1.00 to 1.01)		0.99 (0.96 to 1.02)		1.03 (1.02 to 1.04)
Age†		1.02 (1.01 to 1.02)		1.02 (1.01 to 1.02)		1.03 (0.99 to 1.07)		
Gender: Male		0.98 (0.95 to 1.00)		0.94 (0.91 to 0.96)		1.03 (0.78 to 1.37)		0.89 (0.72 to 1.09)
Diagnosis: RA		1.17 (1.11 to 1.22)		1.17 (1.10 to 1.25)		2.35 (1.56 to 3.68)		1.35 (1.02 to 1.81)
RCS Charlson score*								
0		*Ref*		*Ref*		*Ref*		*Ref*
1		1.17 (1.13 to 1.21)		1.13 (1.09 to 1.18)		0.89 (0.64 to 1.25)		1.27 (0.98 to 1.67)
2+		1.35 (1.26 to 1.44)		1.43 (1.32 to 1.55)		1.49 (0.79 to 3.19)		1.95 (1.31 to 3.03)
IMD quintiles								
1		*Ref*		*Ref*		*Ref*		*Ref*
2		1.04 (1.01 to 1.08)		1.01 (0.97 to 1.05)		1.45 (0.98 to 2.15)		0.92 (0.70 to 1.20)
3		1.07 (1.04 to 1.11)		1.02 (0.97 to 1.06)		1.19 (0.80 to 1.78)		0.85 (0.64 to 1.12)
4		1.07 (1.03 to 1.11)		1.05 (1.00		1.34 (0.90		0.90 (0.65

For peer review only

		to 1.10)	to 2.02)	to 1.25)
5	1.11 (1.06 to 1.16)	1.12 (1.06 to 1.18)	0.94 (0.53 to 1.79)	0.99 (0.70 to 1.45)

Explanatory factors' relative effect (RE) on days of length of stay with 95% confidence interval (CI) from univariable and multivariable regressions. [†]indicates knots for restricted cubic spline. *RA codes were omitted from the calculation of the RCS Charlson score and groups 2 and 3+ combined into 2+.

For peer review only

Table 3. Multivariable regressions for hospital costs

	Primary TKR (RE (95% CI))		Primary THR (RE (95% CI))		Revision TKR (RE (95% CI))		Revision THR (RE (95% CI))	
	Unadjusted	Adjusted	Unadjusted	Adjusted	Unadjusted	Adjusted	Unadjusted	Adjusted
Year of surgery	0.98 (0.97 to 0.98)	0.98 (0.97 to 0.98)	0.98 (0.98 to 0.98)	0.98 (0.98 to 0.98)	0.99 (0.97 to 1.00)	0.99 (0.98 to 1.00)	1.02 (0.99 to 1.04)	1.02 (1.00 to 1.04)
Year of surgery†	1.02 (1.01 to 1.03)	1.02 (1.01 to 1.03)	1.01 (1.01 to 1.01)	1.01 (1.01 to 1.01)			0.97 (0.95 to 1.00)	0.97 (0.94 to 0.99)
Year of surgery††	0.95 (0.91 to 0.99)	0.94 (0.91 to 0.98)						
Age		1.00 (1.00 to 1.00)		1.00 (1.00 to 1.00)		1.01 (1.00 to 1.01)		1.01 (1.00 to 1.01)
Age†		1.00 (1.00 to 1.00)		1.00 (1.00 to 1.01)				
Age††		1.02 (1.00 to 1.03)						
Gender: Male		1.01 (1.00 to 1.01)		0.99 (0.98 to 1.00)		0.99 (0.92 to 1.07)		0.93 (0.86 to 1.00)
Diagnosis: RA		1.04 (1.03 to 1.05)		1.03 (1.01 to 1.04)		1.37 (1.23 to 1.53)		1.13 (1.02 to 1.26)
RCS Charlson score*								
0		*Ref*		*Ref*		*Ref*		*Ref*
1		1.05 (1.05 to 1.06)		1.03 (1.02 to 1.04)		1.04 (0.96 to 1.14)		1.10 (1.00 to 1.21)
2+		1.13 (1.11 to 1.15)		1.10 (1.08 to 1.12)		1.31 (1.10 to 1.58)		1.13 (0.97 to 1.32)
IMD quintiles								
1		*Ref*		*Ref*		*Ref*		*Ref*
2		1.02 (1.01 to 1.03)		1.00 (0.99 to 1.01)		1.05 (0.95 to 1.16)		0.92 (0.84 to 1.02)

For peer review only

3	1.02 (1.01 to 1.03)	1.00 (0.99 to 1.01)	1.06 (0.96 to 1.18)	0.88 (0.80 to 0.98)
4	1.01 (1.00 to 1.02)	1.01 (1.00 to 1.02)	1.08 (0.97 to 1.20)	0.91 (0.81 to 1.03)
5	1.03 (1.02 to 1.04)	1.02 (1.01 to 1.03)	0.94 (0.81 to 1.10)	0.94 (0.82 to 1.08)

Explanatory factors' relative effect (RE) of diagnosis on hospital costs (in 2017 GBP) with 95% confidence interval (CI) from univariable and multivariable regressions. †indicates knots for restricted cubic spline. *RA codes were omitted from the calculation of the RCS Charlson score and groups 2 and 3+ combined into 2+.

For peer review only

Figure 1. Study inclusion flow chart

Figure 2. Trends in length of stay. Estimated effect of year of surgery on length of stay (in days). TKR: total knee replacement, THR: total hip replacement.

Figure 3. Trends in hospital costs. Estimated effect of year of surgery on hospital costs (in 2017 GBP). TKR: total knee replacement, THR: total hip replacement.

For peer review only

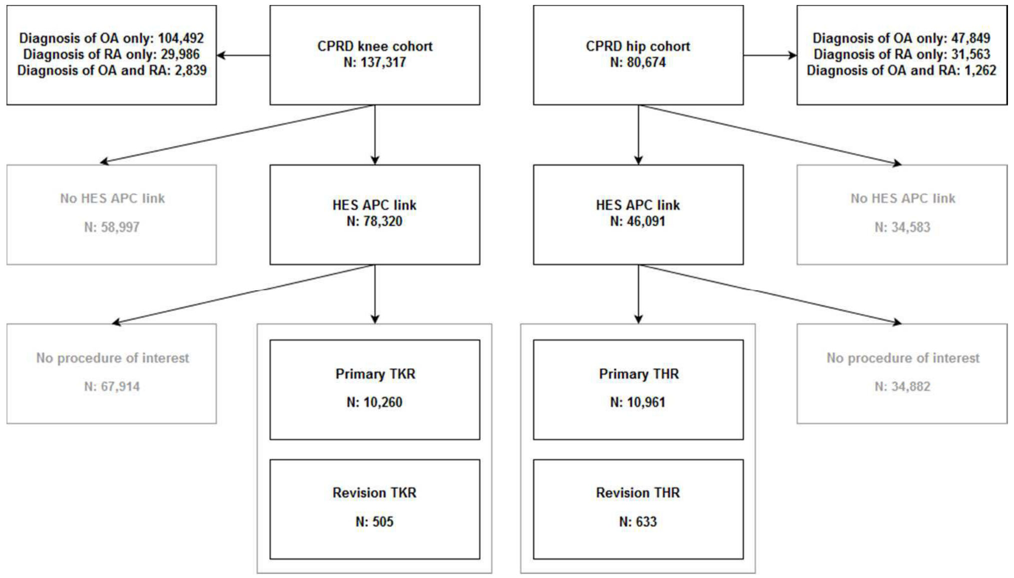

Figure 1. Study inclusion flow chart

353x201mm (72 x 72 DPI)

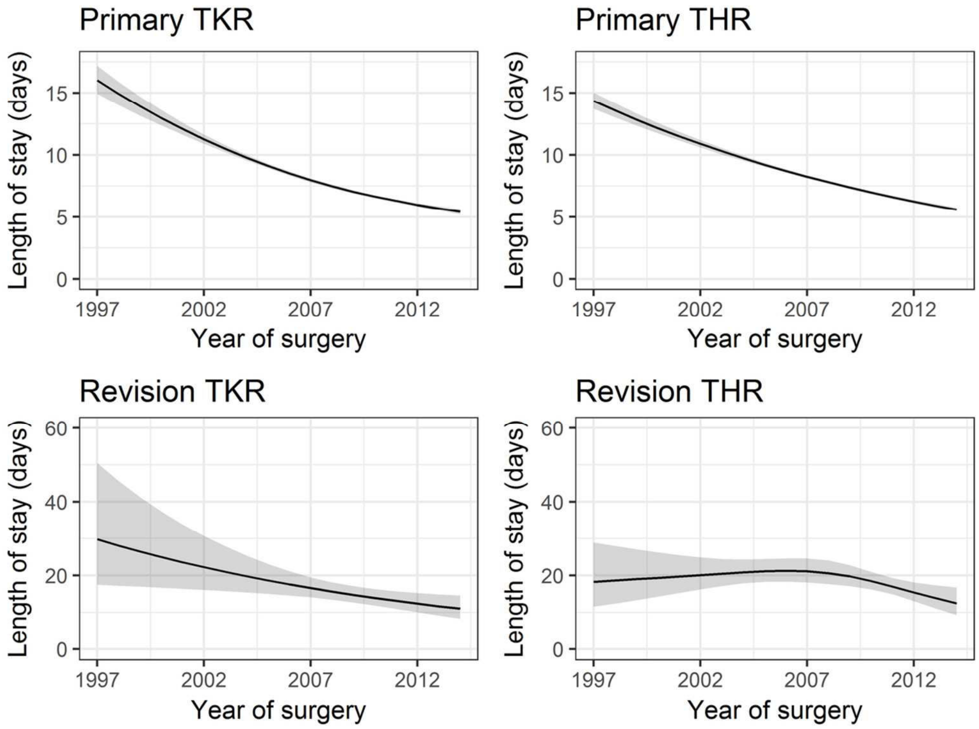

Figure 2. Trends in length of stay. Estimated effect of year of surgery on length of stay (in days). TKR: total knee replacement, THR: total hip replacement.

114x85mm (300 x 300 DPI)

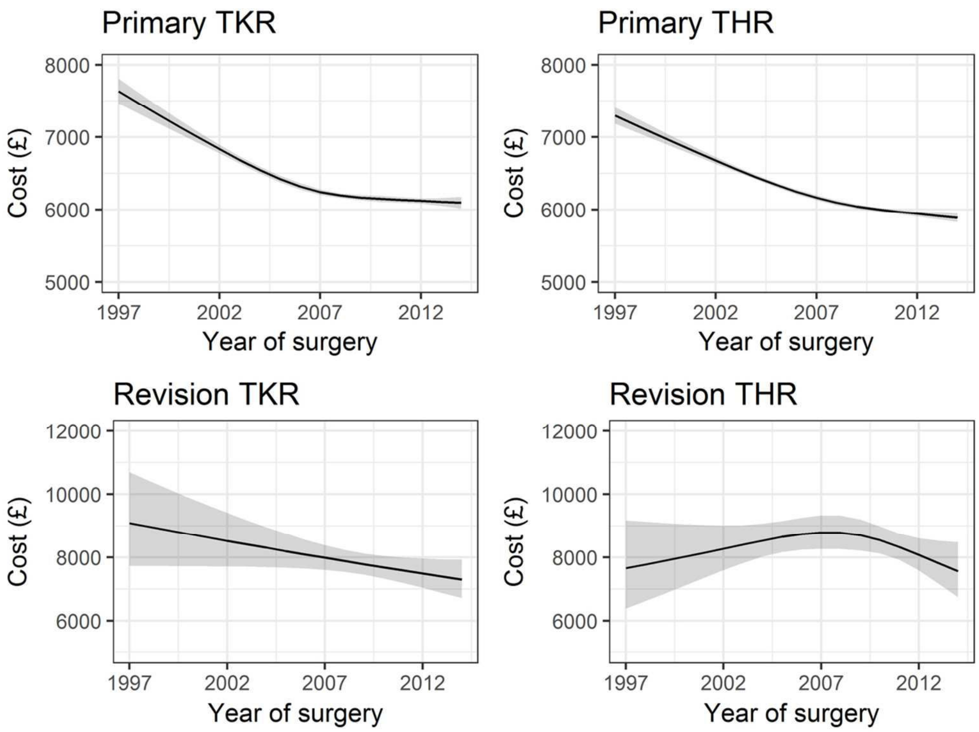

Figure 3. Trends in hospital costs. Estimated effect of year of surgery on hospital costs (in 2017 GBP). TKR: total knee replacement, THR: total hip replacement.

114x85mm (300 x 300 DPI)

For peer review only

APPENDIX

Table of Contents

Patient characteristics by year of surgery 2

Primary total knee replacement 2

Primary total hip replacement 4

Revision total knee replacement 6

Revision total hip replacement 8

Length of stay by year of surgery 10

Hospital costs by year of surgery 12

Partial effect of age on length of stay and costs 14

Partial effect of age at surgery on length of stay from multivariable regressions. Predicted length of stay by age while other explanatory variables are held fixed at their average (median if continuous and mode if categorical). TKR: total knee replacement, THR: total hip replacement. 14

Partial effect of age at surgery on hospital costs (in 2017 GBP) from multivariable regressions. Predicted cost by age while other explanatory variables are held fixed at their average (median if continuous and mode if categorical). TKR: total knee replacement, THR: total hip replacement. 14

For peer review only - http://bmjopen.bmj.com/site/about/guidelines.xhtml

PATIENT CHARACTERISTICS BY YEAR OF SURGERY

Primary total knee replacement

Year of surgery	n	Age (mean (sd))	Gender: Male (n (%))	Rheumatoid arthritis (n (%))	RCS Charlson score (n (%)) 0	1	2	3+	IMD quintiles (n (%)) 1	2	3	4	5
1997	45	72 (9)	18 (40%)	8 (18%)	43 (96%)	2 (4%)	0 (0%)	0 (0%)	7 (16%)	9 (20%)	13 (29%)	8 (18%)	8 (18%)
1998	64	72 (10)	23 (36%)	12 (19%)	57 (89%)	5 (8%)	1 (0%)	1 (0%)	11 (17%)	14 (22%)	16 (25%)	14 (22%)	9 (14%)
1999	114	71 (9)	45 (39%)	6 (5%)	98 (86%)	14 (12%)	2 (0%)	0 (0%)	18 (16%)	32 (28%)	17 (15%)	30 (27%)	16 (14%)
2000	115	72 (9)	55 (48%)	16 (14%)	97 (84%)	16 (14%)	1 (0%)	1 (0%)	22 (19%)	31 (27%)	19 (17%)	21 (18%)	22 (19%)
2001	170	71 (9)	76 (45%)	18 (11%)	152 (89%)	16 (9%)	2 (0%)	0 (0%)	40 (24%)	34 (20%)	43 (25%)	31 (18%)	22 (13%)
2002	268	70 (10)	116 (43%)	28 (10%)	237 (88%)	28 (10%)	3 (0%)	0 (0%)	68 (25%)	67 (25%)	61 (23%)	39 (15%)	33 (12%)
2003	403	71 (9)	173 (43%)	40 (10%)	344 (85%)	51 (13%)	7 (0%)	1 (0%)	95 (24%)	96 (24%)	101 (25%)	64 (16%)	47 (12%)
2004	545	70 (9)	216 (40%)	60 (11%)	458 (84%)	78 (14%)	7 (0%)	2 (0%)	132 (24%)	116 (21%)	146 (27%)	102 (19%)	48 (9%)
2005	605	71 (9)	255 (42%)	60 (10%)	509 (84%)	83 (14%)	11 (0%)	2 (0%)	135 (22%)	129 (21%)	140 (23%)	137 (23%)	62 (10%)
2006	735	71 (9)	315 (43%)	75 (10%)	584 (79%)	135 (18%)	13 (0%)	3 (0%)	157 (21%)	168 (23%)	187 (25%)	142 (19%)	81 (11%)
2007	912	70 (9)	371 (41%)	83 (9%)	742 (81%)	141 (15%)	28 (0%)	1 (0%)	201 (22%)	207 (23%)	204 (22%)	190 (21%)	110 (12%)
2008	981	70 (9)	432 (44%)	73 (7%)	775 (79%)	177 (18%)	28 (0%)	1 (0%)	229 (23%)	243 (25%)	204 (21%)	188 (19%)	115 (12%)
2009	1045	69 (9)	463 (44%)	71 (7%)	773	234	29	9	229	282	247	190	96

For peer review only

					(74%)	(22%)	(0%)	(0%)	(22%)	(27%)	(24%)	(18%)	(9%)
2010	1009	70 (9)	440 (44%)	72 (7%)	744 (74%)	209 (21%)	48 (0%)	8 (0%)	253 (25%)	249 (25%)	230 (23%)	176 (17%)	101 (10%)
2011	1024	69 (9)	461 (45%)	78 (8%)	715 (70%)	249 (24%)	54 (1%)	6 (0%)	229 (22%)	236 (23%)	250 (24%)	200 (20%)	109 (11%)
2012	1018	69 (9)	444 (44%)	69 (7%)	690 (68%)	273 (27%)	48 (0%)	7 (0%)	236 (23%)	269 (26%)	217 (21%)	182 (18%)	113 (11%)
2013	967	70 (9)	416 (43%)	65 (7%)	633 (65%)	272 (28%)	51 (0%)	11 (0%)	216 (22%)	251 (26%)	212 (22%)	184 (19%)	103 (11%)
2014	240	69 (9)	107 (45%)	17 (7%)	153 (64%)	66 (28%)	20 (0%)	1 (0%)	52 (22%)	54 (22%)	76 (32%)	32 (13%)	26 (11%)

For peer review only

For peer review only - http://bmjopen.bmj.com/site/about/guidelines.xhtml

Primary total hip replacement

Year of surgery	n	Age (mean (sd))	Gender: Male (n (%))	Rheumatoid arthritis (n (%))	RCS Charlson score (n (%))				IMD quintiles (n (%))				
					0	1	2	3+	1	2	3	4	5
1997	78	69 (10)	32 (41%)	5 (6%)	73 (94%)	5 (6%)	0 (0%)	0 (0%)	21 (27%)	15 (19%)	20 (26%)	16 (21%)	6 (8%)
1998	128	69 (10)	42 (33%)	8 (6%)	120 (94%)	7 (5%)	1 (0%)	0 (0%)	27 (21%)	26 (20%)	34 (27%)	23 (18%)	18 (14%)
1999	195	69 (12)	80 (41%)	11 (6%)	172 (88%)	22 (11%)	1 (0%)	0 (0%)	46 (24%)	47 (24%)	47 (24%)	32 (16%)	23 (12%)
2000	242	70 (10)	104 (43%)	6 (2%)	217 (90%)	22 (9%)	3 (0%)	0 (0%)	57 (24%)	79 (33%)	50 (21%)	37 (15%)	19 (8%)
2001	317	68 (10)	137 (43%)	14 (4%)	284 (90%)	28 (9%)	3 (0%)	2 (0%)	81 (26%)	87 (27%)	65 (21%)	54 (17%)	30 (9%)
2002	446	69 (10)	163 (37%)	27 (6%)	396 (89%)	44 (10%)	6 (0%)	0 (0%)	104 (23%)	115 (26%)	105 (24%)	75 (17%)	47 (11%)
2003	563	69 (10)	244 (43%)	35 (6%)	490 (87%)	66 (12%)	7 (0%)	0 (0%)	153 (27%)	140 (25%)	130 (23%)	99 (18%)	41 (7%)
2004	648	70 (10)	268 (41%)	36 (6%)	546 (84%)	89 (14%)	12 (0%)	1 (0%)	181 (28%)	166 (26%)	142 (22%)	100 (15%)	59 (9%)
2005	760	68 (11)	312 (41%)	51 (7%)	626 (82%)	114 (15%)	18 (0%)	2 (0%)	178 (23%)	190 (25%)	162 (21%)	153 (20%)	75 (10%)
2006	809	69 (11)	318 (39%)	48 (6%)	656 (81%)	131 (16%)	20 (0%)	2 (0%)	199 (25%)	210 (26%)	166 (21%)	150 (19%)	83 (10%)
2007	922	69 (11)	393 (43%)	56 (6%)	743 (81%)	154 (17%)	24 (0%)	1 (0%)	247 (27%)	240 (26%)	198 (21%)	167 (18%)	70 (8%)
2008	942	69 (11)	403 (43%)	52 (6%)	750 (80%)	158 (17%)	28 (0%)	6 (0%)	226 (24%)	227 (24%)	212 (23%)	181 (19%)	96 (10%)
2009	963	69 (11)	410 (43%)	46 (5%)	766 (80%)	166 (17%)	25 (0%)	6 (0%)	248 (26%)	259 (27%)	216 (22%)	166 (17%)	72 (7%)

For peer review only

For peer review only - http://bmjopen.bmj.com/site/about/guidelines.xhtml

2010	943	69 (11)	412 (44%)	57 (6%)	688 (73%)	222 (24%)	26 (0%)	7 (0%)	219 (23%)	250 (27%)	221 (23%)	154 (16%)	99 (10%)
2011	954	69 (10)	376 (39%)	64 (7%)	710 (74%)	191 (20%)	42 (0%)	11 (0%)	238 (25%)	256 (27%)	225 (24%)	170 (18%)	65 (7%)
2012	963	69 (10)	369 (38%)	66 (7%)	679 (71%)	224 (23%)	47 (0%)	13 (0%)	249 (26%)	226 (23%)	238 (25%)	166 (17%)	84 (9%)
2013	879	68 (11)	370 (42%)	43 (5%)	614 (70%)	213 (24%)	40 (0%)	12 (0%)	245 (28%)	215 (24%)	199 (23%)	145 (17%)	74 (8%)
2014	209	70 (10)	92 (44%)	14 (7%)	147 (70%)	52 (25%)	8 (0%)	2 (0%)	60 (29%)	46 (22%)	49 (23%)	35 (17%)	19 (9%)

For peer review only

For peer review only - http://bmjopen.bmj.com/site/about/guidelines.xhtml

Revision total knee replacement

Year of surgery	n	Age (mean (sd))	Gender: Male (n (%))	Rheumatoid arthritis (n (%))	RCS Charlson score (n (%))				IMD quintiles (n (%))				
					0	1	2	3+	1	2	3	4	5
1997	2	74 (1)	1 (50%)	0 (0%)	2 (100%)	0 (0%)	0 (0%)	0 (0%)	1 (50%)	1 (50%)	0 (0%)	0 (0%)	0 (0%)
1998	3	78 (2)	1 (33%)	2 (67%)	3 (100%)	0 (0%)	0 (0%)	0 (0%)	2 (67%)	0 (0%)	0 (0%)	1 (33%)	0 (0%)
1999	1	64 (-)	0 (0%)	1 (100%)	1 (100%)	0 (0%)	0 (0%)	0 (0%)	0 (0%)	0 (0%)	0 (0%)	1 (100%)	0 (0%)
2000	3	60 (19)	1 (33%)	3 (100%)	3 (100%)	0 (0%)	0 (0%)	0 (0%)	0 (0%)	1 (33%)	1 (33%)	0 (0%)	1 (33%)
2001	4	68 (13)	3 (75%)	0 (0%)	3 (75%)	1 (25%)	0 (0%)	0 (0%)	0 (0%)	2 (50%)	0 (0%)	1 (25%)	1 (25%)
2002	9	74 (9)	6 (67%)	1 (11%)	9 (100%)	0 (0%)	0 (0%)	0 (0%)	1 (11%)	6 (67%)	1 (11%)	1 (11%)	0 (0%)
2003	20	71 (11)	12 (60%)	3 (15%)	16 (80%)	4 (20%)	0 (0%)	0 (0%)	5 (25%)	2 (10%)	7 (35%)	5 (25%)	1 (5%)
2004	23	67 (10)	13 (57%)	4 (17%)	17 (74%)	4 (17%)	2 (0%)	0 (0%)	2 (9%)	8 (35%)	8 (35%)	4 (17%)	1 (4%)
2005	23	69 (11)	10 (43%)	4 (17%)	20 (87%)	3 (13%)	0 (0%)	0 (0%)	8 (35%)	4 (17%)	2 (9%)	8 (35%)	1 (4%)
2006	35	68 (9)	15 (43%)	6 (17%)	26 (74%)	8 (23%)	1 (0%)	0 (0%)	11 (31%)	8 (23%)	8 (23%)	6 (17%)	2 (6%)
2007	41	68 (10)	22 (54%)	4 (10%)	31 (76%)	5 (12%)	4 (1%)	1 (0%)	9 (22%)	5 (12%)	10 (24%)	13 (32%)	4 (10%)
2008	57	68 (10)	28 (49%)	9 (16%)	39 (68%)	17 (30%)	1 (0%)	0 (0%)	12 (21%)	12 (21%)	13 (23%)	11 (19%)	9 (16%)
2009	60	72 (10)	24 (40%)	7 (12%)	49 (82%)	10 (17%)	1 (0%)	0 (0%)	15 (25%)	17 (28%)	11 (18%)	14 (23%)	3 (5%)
2010	59	69 (10)	26 (44%)	8 (14%)	36	20	2	1	15	15	15	10	4 (7%)

For peer review only

					(61%)	(34%)	(0%)	(0%)	(25%)	(25%)	(25%)	(17%)	
2011	58	70 (10)	26 (45%)	6 (10%)	38 (66%)	18 (31%)	2 (0%)	0 (0%)	14 (24%)	16 (28%)	16 (28%)	9 (16%)	3 (5%)
2012	55	69 (10)	26 (47%)	3 (5%)	36 (65%)	15 (27%)	4 (1%)	0 (0%)	19 (35%)	12 (22%)	10 (18%)	12 (22%)	2 (4%)
2013	33	71 (9)	14 (42%)	3 (9%)	20 (61%)	9 (27%)	4 (1%)	0 (0%)	7 (21%)	9 (27%)	10 (30%)	6 (18%)	1 (3%)
2014	18	69 (9)	5 (28%)	1 (6%)	12 (67%)	5 (28%)	0 (0%)	1 (0%)	7 (39%)	2 (11%)	4 (22%)	3 (17%)	2 (11%)

For peer review only

For peer review only - http://bmjopen.bmj.com/site/about/guidelines.xhtml

Revision total hip replacement

Year of surgery	n	Age (mean (sd))	Gender: Male (n (%))	Rheumatoid arthritis (n (%))	RCS Charlson score (n (%)) 0	1	2	3+	IMD quintiles (n (%)) 1	2	3	4	5
1997	2	74 (11)	1 (50%)	1 (50%)	2 (100%)	0 (0%)	0 (0%)	0 (0%)	2 (100%)	0 (0%)	0 (0%)	0 (0%)	0 (0%)
1998	6	72 (8)	2 (33%)	0 (0%)	6 (100%)	0 (0%)	0 (0%)	0 (0%)	4 (67%)	2 (33%)	0 (0%)	0 (0%)	0 (0%)
1999	8	70 (14)	3 (38%)	1 (12%)	8 (100%)	0 (0%)	0 (0%)	0 (0%)	3 (38%)	3 (38%)	1 (12%)	1 (12%)	0 (0%)
2000	14	72 (11)	9 (64%)	2 (14%)	11 (79%)	3 (21%)	0 (0%)	0 (0%)	5 (36%)	1 (7%)	5 (36%)	1 (7%)	2 (14%)
2001	14	69 (10)	5 (36%)	2 (14%)	14 (100%)	0 (0%)	0 (0%)	0 (0%)	3 (21%)	2 (14%)	3 (21%)	5 (36%)	1 (7%)
2002	8	73 (11)	4 (50%)	2 (25%)	7 (88%)	1 (12%)	0 (0%)	0 (0%)	4 (50%)	1 (12%)	2 (25%)	0 (0%)	1 (12%)
2003	22	68 (11)	8 (36%)	7 (32%)	21 (95%)	1 (5%)	0 (0%)	0 (0%)	9 (41%)	5 (23%)	4 (18%)	2 (9%)	2 (9%)
2004	35	70 (10)	15 (43%)	6 (17%)	29 (83%)	6 (17%)	0 (0%)	0 (0%)	16 (46%)	7 (20%)	5 (14%)	3 (9%)	4 (11%)
2005	41	73 (10)	19 (46%)	7 (17%)	32 (78%)	9 (22%)	0 (0%)	0 (0%)	10 (24%)	9 (22%)	8 (20%)	8 (20%)	6 (15%)
2006	33	66 (12)	17 (52%)	5 (15%)	26 (79%)	3 (9%)	4 (1%)	0 (0%)	12 (36%)	8 (24%)	9 (27%)	1 (3%)	3 (9%)
2007	54	70 (10)	22 (41%)	5 (9%)	43 (80%)	10 (19%)	1 (0%)	0 (0%)	21 (39%)	16 (30%)	10 (19%)	3 (6%)	4 (7%)
2008	57	69 (11)	23 (40%)	8 (14%)	45 (79%)	10 (18%)	1 (0%)	1 (0%)	20 (35%)	12 (21%)	11 (19%)	10 (18%)	4 (7%)
2009	54	70 (13)	19 (35%)	9 (17%)	40 (74%)	12 (22%)	1 (0%)	1 (0%)	15 (28%)	18 (33%)	9 (17%)	9 (17%)	3 (6%)

For peer review only - http://bmjopen.bmj.com/site/about/guidelines.xhtml

2010	71	67 (12)	33 (46%)	7 (10%)	54 (76%)	14 (20%)	3 (0%)	0 (0%)	23 (32%)	20 (28%)	11 (15%)	9 (13%)	8 (11%)
2011	67	73 (11)	29 (43%)	4 (6%)	51 (76%)	7 (10%)	8 (1%)	1 (0%)	16 (24%)	14 (21%)	15 (22%)	12 (18%)	10 (15%)
2012	71	71 (9)	28 (39%)	14 (20%)	47 (66%)	15 (21%)	7 (1%)	2 (0%)	25 (35%)	15 (21%)	18 (25%)	10 (14%)	3 (4%)
2013	66	71 (9)	24 (36%)	10 (15%)	41 (62%)	17 (26%)	6 (1%)	2 (0%)	18 (27%)	20 (30%)	13 (20%)	8 (12%)	7 (11%)
2014	9	76 (14)	3 (33%)	1 (11%)	5 (56%)	3 (33%)	1 (0%)	0 (0%)	2 (22%)	2 (22%)	3 (33%)	1 (11%)	1 (11%)

For peer review only - http://bmjopen.bmj.com/site/about/guidelines.xhtml

For peer review only

LENGTH OF STAY BY YEAR OF SURGERY

Year	TKR		THR		TKR revision		THR revision	
	N	Mean (se)	N	Mean (se)	N	Mean (se)	N	Mean (se)
1997	45	18.89 (3.12)	78	14.28 (1.47)	2	12.5 (2.5)	3	27.33 (5.67)
1998	64	14.77 (0.78)	128	12.49 (0.42)	3	20 (2.08)	6	14.83 (1.08)
1999	114	12.79 (0.51)	195	12.1 (0.39)	1	18 (-)	8	18.75 (6.21)
2000	116	13.25 (1.09)	242	12.54 (0.64)	3	74 (59.03)	14	15 (1.79)
2001	170	12.13 (0.48)	319	11.97 (0.5)	4	15.25 (6.65)	14	21.86 (5.16)
2002	268	11.12 (0.42)	447	10.89 (0.26)	9	24 (7.11)	8	32.12 (12.92)
2003	403	10.32 (0.22)	561	10.3 (0.25)	21	21.29 (4.61)	23	22.48 (5.47)
2004	545	9.58 (0.3)	647	9.82 (0.29)	24	16.54 (3.61)	35	20.49 (3.42)
2005	604	9.11 (0.38)	761	9.33 (0.24)	22	19.64 (6.52)	40	21.48 (4.87)
2006	735	8.75 (0.27)	812	8.79 (0.22)	36	15.08 (3.35)	36	14.11 (2.87)
2007	913	8.1 (0.2)	923	8.25 (0.26)	41	13.41 (3.01)	58	15.28 (2.28)
2008	982	7.49 (0.24)	940	7.94 (0.3)	56	17.45 (4.1)	53	30.66 (5.38)
2009	1043	7.14 (0.21)	964	7.09 (0.18)	60	15.22 (2.74)	53	22.28 (4.33)
2010	1009	6.43 (0.11)	941	7.02 (0.17)	61	18.57 (5.55)	70	14.94 (3.05)
2011	1024	6.12 (0.1)	954	6.27 (0.13)	57	9.86 (1.35)	66	17.12 (2.55)
2012	1018	6.03 (0.16)	963	6.66 (0.3)	53	11.19 (2.03)	74	17.47 (3.05)
2013	967	5.89 (0.12)	877	5.83 (0.14)	35	15.4 (3.51)	63	11.87 (1.31)

For peer review only

2014	240	5.13 (0.15)	209	5.6 (0.3)	17	6.06 (0.8)	9	15.33 (4.78)

Number of study participants (N) with mean length of stay with standard error (se) by year. THR: total hip replacement, TKR: total knee replacement

For peer review only

For peer review only - http://bmjopen.bmj.com/site/about/guidelines.xhtml

HOSPITAL COSTS BY YEAR OF SURGERY

Year	Primary TKR		Primary THR		Revision TKR		Revision THR	
	N	Mean (se)	N	Mean (se)	N	Mean (se)	N	Mean (se)
1997	45	8516.45 (708.81)	78	7428.29 (359.68)	2	7562 (-)	3	9500.67 (2200.33)
1998	64	7590.65 (180.75)	128	7101.09 (98.33)	3	9677 (-)	6	7610.83 (362.58)
1999	114	7140.98 (128.49)	195	6921.47 (73.94)	1	8267 (-)	8	7244 (989.48)
2000	116	7168.41 (241.34)	242	6968.52 (103.49)	3	7562 (575.63)	14	7540.93 (500.08)
2001	170	6977.79 (100.8)	319	6839.25 (94.39)	4	6239.75 (1392.38)	14	8086.08 (670.15)
2002	268	6774.28 (89.49)	447	6669.12 (53.2)	9	9339.75 (1769.17)	8	10039.57 (2172.19)
2003	403	6590.4 (45.92)	561	6564.01 (54.2)	21	8544.84 (990.89)	23	8855 (907.28)
2004	545	6477.54 (68.11)	647	6417.76 (44.49)	24	7653.09 (493.94)	35	8442.61 (579.42)
2005	604	6393.04 (68.77)	761	6379.89 (53.06)	22	8306.64 (1423.54)	40	8688.02 (830.29)
2006	735	6401.5 (60.82)	812	6263 (40.8)	36	8026.77 (680.29)	36	7786.17 (468.81)
2007	913	6289.42 (42.25)	923	6155.92 (39.35)	41	8259.78 (595.51)	58	7942.64 (407.56)
2008	982	6194.13 (42.4)	940	6152.11 (58.67)	56	8249.2 (698.63)	53	10707.98 (989.17)

For peer review only

2009	1043	6202.38 (44.82)	964	5968.21 (24.92)	60	8056.98 (573.84)	53	8424.12 (614.65)
2010	1009	6107.99 (20.9)	941	6013.59 (31.66)	61	7829.78 (433.18)	70	8125.48 (608.8)
2011	1024	6108.19 (20.4)	954	5920.3 (24.32)	57	7130.88 (163.09)	66	8015.88 (383.55)
2012	1018	6136.84 (30.12)	963	6016.33 (47.13)	53	6906.06 (219.48)	74	8773.54 (596.93)
2013	967	6131.57 (22.07)	877	5900.26 (23.51)	35	8177.89 (737.29)	63	7523.76 (213.64)
2014	240	6053.24 (32.19)	209	5870.64 (50.52)	17	6761.94 (81.42)	9	7071.88 (615.47)

Number of study participants (N) with mean cost with standard error (se) by year. THR: total hip replacement, TKR: total knee replacement

For peer review only

PARTIAL EFFECT OF AGE ON LENGTH OF STAY AND COSTS

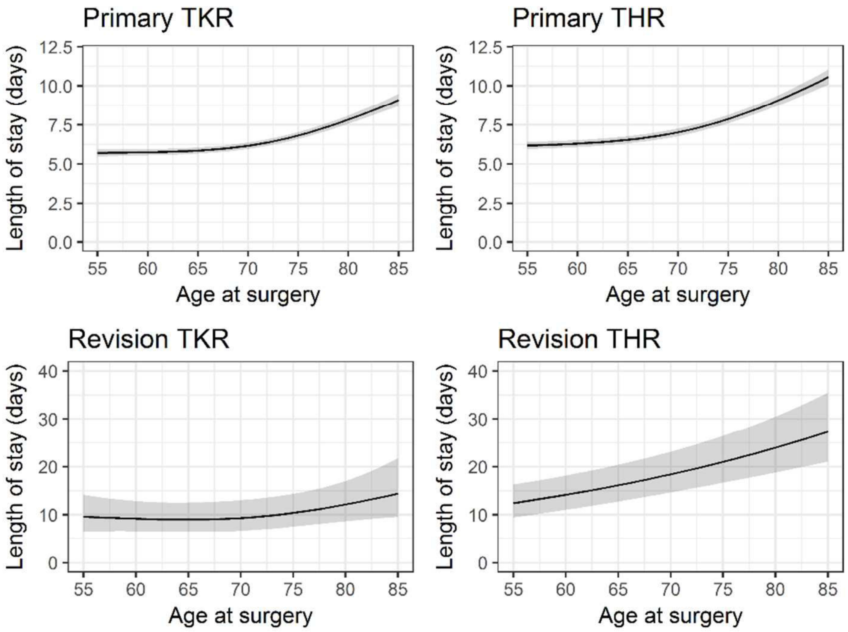

Partial effect of age at surgery on length of stay from multivariable regressions. Predicted length of stay by age while other explanatory variables are held fixed at their average (median if continuous and mode if categorical). TKR: total knee replacement, THR: total hip replacement.

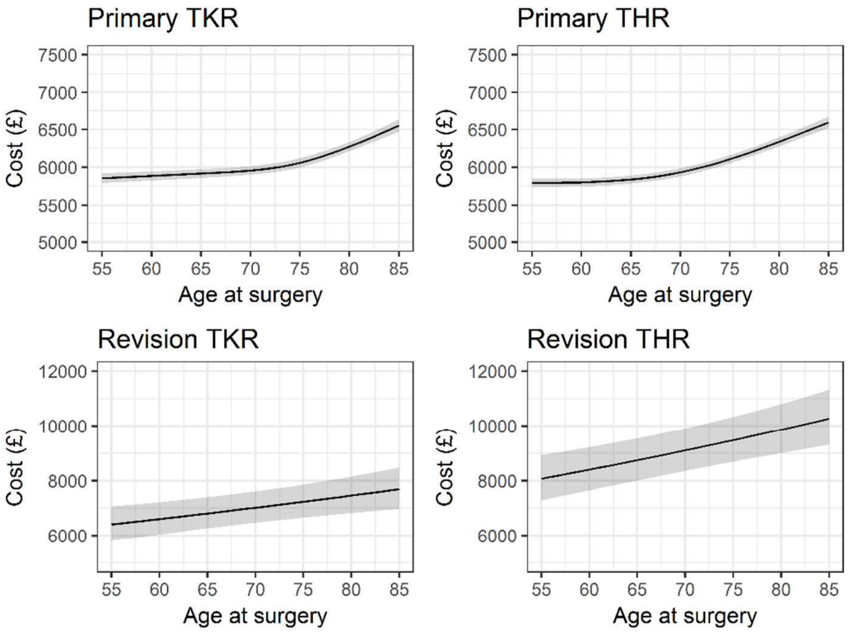

Partial effect of age at surgery on hospital costs (in 2017 GBP) from multivariable regressions. Predicted cost by age while other explanatory variables are held fixed at their average (median if continuous and mode if categorical). TKR: total knee replacement, THR: total hip replacement.

For peer review only - http://bmjopen.bmj.com/site/about/guidelines.xhtml

STROBE Statement—checklist of items that should be included in reports of observational studies

	Item No	Recommendation	Line No.
**Title and abstract**	1	(*a*) Indicate the study's design with a commonly used term in the title or the abstract	1
		(*b*) Provide in the abstract an informative and balanced summary of what was done and what was found	27-59
**Introduction**			
Background/rationale	2	Explain the scientific background and rationale for the investigation being reported	74-96
Objectives	3	State specific objectives, including any prespecified hypotheses	97-101
**Methods**			
Study design	4	Present key elements of study design early in the paper	103-111
Setting	5	Describe the setting, locations, and relevant dates, including periods of recruitment, exposure, follow-up, and data collection	122-137
Participants	6	(*a*) *Cohort study*—Give the eligibility criteria, and the sources and methods of selection of participants. Describe methods of follow-up<br>*Case-control study*—Give the eligibility criteria, and the sources and methods of case ascertainment and control selection. Give the rationale for the choice of cases and controls<br>*Cross-sectional study*—Give the eligibility criteria, and the sources and methods of selection of participants	153-167
		(*b*) *Cohort study*—For matched studies, give matching criteria and number of exposed and unexposed<br>*Case-control study*—For matched studies, give matching criteria and the number of controls per case	
Variables	7	Clearly define all outcomes, exposures, predictors, potential confounders, and effect modifiers. Give diagnostic criteria, if applicable	138-161
Data sources/ measurement	8*	For each variable of interest, give sources of data and details of methods of assessment (measurement). Describe comparability of assessment methods if there is more than one group	138-161
Bias	9	Describe any efforts to address potential sources of bias	
Study size	10	Explain how the study size was arrived at	
Quantitative variables	11	Explain how quantitative variables were handled in the analyses. If applicable, describe which groupings were chosen and why	171-177
Statistical methods	12	(*a*) Describe all statistical methods, including those used to control for confounding	163-182
		(*b*) Describe any methods used to examine subgroups and interactions	
		(*c*) Explain how missing data were addressed	183-186
		(*d*) *Cohort study*—If applicable, explain how loss to follow-up was addressed<br>*Case-control study*—If applicable, explain how matching of cases and controls was addressed<br>*Cross-sectional study*—If applicable, describe analytical methods taking	

For peer review only

		account of sampling strategy	
		(*e*) Describe any sensitivity analyses	
**Results**			
Participants	13*	(a) Report numbers of individuals at each stage of study—eg numbers potentially eligible, examined for eligibility, confirmed eligible, included in the study, completing follow-up, and analysed	Figure 1, 193-203
		(b) Give reasons for non-participation at each stage	Figure 1
		(c) Consider use of a flow diagram	Figure 1
Descriptive data	14*	(a) Give characteristics of study participants (eg demographic, clinical, social) and information on exposures and potential confounders	Table 1
		(b) Indicate number of participants with missing data for each variable of interest	Table 1
		(c) *Cohort study*—Summarise follow-up time (eg, average and total amount)	
Outcome data	15*	*Cohort study*—Report numbers of outcome events or summary measures over time	Table 1, Appendix
		Case-control study—Report numbers in each exposure category, or summary measures of exposure	
		Cross-sectional study—Report numbers of outcome events or summary measures	
Main results	16	(*a*) Give unadjusted estimates and, if applicable, confounder-adjusted estimates and their precision (eg, 95% confidence interval). Make clear which confounders were adjusted for and why they were included	Tables 2 and 3,
		(*b*) Report category boundaries when continuous variables were categorized	
		(*c*) If relevant, consider translating estimates of relative risk into absolute risk for a meaningful time period	205-210,
Other analyses	17	Report other analyses done—eg analyses of subgroups and interactions, and sensitivity analyses	
**Discussion**			
Key results	18	Summarise key results with reference to study objectives	243-265
Limitations	19	Discuss limitations of the study, taking into account sources of potential bias or imprecision. Discuss both direction and magnitude of any potential bias	267-282
Interpretation	20	Give a cautious overall interpretation of results considering objectives, limitations, multiplicity of analyses, results from similar studies, and other relevant evidence	289-332
Generalisability	21	Discuss the generalisability (external validity) of the study results	
**Other information**			
Funding	22	Give the source of funding and the role of the funders for the present study and, if applicable, for the original study on which the present article is based	340-344

*Give information separately for cases and controls in case-control studies and, if applicable, for exposed and unexposed groups in cohort and cross-sectional studies.

Note: An Explanation and Elaboration article discusses each checklist item and gives methodological background and published examples of transparent reporting. The STROBE checklist is best used in conjunction with this article (freely available on the Web sites of PLoS Medicine at http://www.plosmedicine.org/, Annals of Internal Medicine at

For peer review only

For peer review only - http://bmjopen.bmj.com/site/about/guidelines.xhtml

http://www.annals.org/, and Epidemiology at http://www.epidem.com/). Information on the STROBE Initiative is available at www.strobe-statement.org.

For peer review only

BMJ Open

Trends and determinants of length of stay and hospital reimbursement following knee and hip replacement: evidence from linked primary care and NHS hospital records from 1997 to 2014

Journal:	*BMJ Open*
Manuscript ID	bmjopen-2017-019146.R1
Article Type:	Research
Date Submitted by the Author:	25-Oct-2017
Complete List of Authors:	Burn, Edward; University of Oxford Nuffield Department of Orthopaedics Rheumatology and Musculoskeletal Sciences<br>Edwards, Christopher; NIHR Wellcome Trust Clinical Research Facility, University Hospital Southampton<br>Murray, David; University of Oxford, Nuffield Department of Orthopaedics, Rheumatology & Musculoskeletal Sciences<br>Silman, Alan; University of Oxford Nuffield Department of Orthopaedics Rheumatology and Musculoskeletal Sciences<br>Cooper, Cyrus; University of Southampton and Southampton University Hospitals NHS Trust, MRC Lifecourse Epidemiology Unit; University of Oxford Nuffield Department of Orthopaedics Rheumatology and Musculoskeletal Sciences<br>Arden, Nigel; Nuffield Department of Orthopaedics; MRC Lifecourse Epidemiology Unit, Southampton University<br>Pinedo-Villanueva, Rafael; University of Oxford Nuffield Department of Orthopaedics Rheumatology and Musculoskeletal Sciences, Nuffield Department of Orthopaedics Rheumatology and Musculoskeletal Sciences; MRC Lifecourse Epidemiology Unit, Southampton University<br>Prieto-Alhambra, Daniel; University of Oxford Nuffield Department of Orthopaedics Rheumatology and Musculoskeletal Sciences; GREMPAL Research Group, Idiap Jordi Gol and CIBERFes, Universitat Autonoma de Barcelona and Instituto de Salud Carlos III
**Primary Subject Heading**:	Health services research
Secondary Subject Heading:	Health economics
Keywords:	Joint Replacement, Knee Arthroplasty, Hip Arthroplasty, Length of Stay, Costs

SCHOLARONE™
Manuscripts

Trends and determinants of length of stay and hospital reimbursement following knee and hip replacement: evidence from linked primary care and NHS hospital records from 1997 to 2014

Authors:

Edward Burn, MSc[1], Christopher J Edwards, FRCP, [2] David W Murray, MD,[1] Alan Silman, FMedSci,[1] Cyrus Cooper, FMedSci,[1,3] Nigel K Arden, FRCP, [1,3] Rafael Pinedo-Villanueva, PhD, [1,3] Daniel Prieto-Alhambra, PhD[1,4]

Affiliations:

[1]Nuffield Department of Orthopaedics, Rheumatology and Musculoskeletal Sciences, University of Oxford

2 NIHR Wellcome Trust Clinical Research Facility, University Hospital Southampton, Southampton, UK

3 MRC Lifecourse Epidemiology Unit, Southampton University, Southampton, UK

4 GREMPAL Research Group, Idiap Jordi Gol and CIBERFes, Universitat Autonoma de Barcelona and Instituto de Salud Carlos III, Barcelona, Spain

Correspondence to: Rafael Pinedo-Villanueva (rafael.pinedo@ndorms.ox.ac.uk)

Key words: Joint Replacement, Knee Arthroplasty, Hip Arthroplasty, Length of Stay, Costs

For peer review only

ABSTRACT

Objectives

To measure changes in length of stay following total knee and hip replacement (TKR and THR) between 1997 and 2014 and estimate the impact on hospital reimbursement, all else being equal. Further, to assess the degree to which observed trends can be explained by improved efficiency or changes in patient profiles.

Design

Cross-sectional study using routinely collected data.

Setting

NHS primary care records from 1995 to 2014 in the Clinical Practice Research Datalink (CPRD) were linked to hospital inpatient data from 1995 to 2014 in Hospital Episode Statistics Admitted Patient Care (HES APC)

Participants

Study participants had a diagnosis of osteoarthritis (OA) or rheumatoid arthritis (RA).

Interventions

Primary TKR, primary THR, revision TKR, and revision THR.

Primary outcome measures

Length of stay and hospital reimbursement.

Results

10,260 primary TKR, 10,961 primary THR, 505 revision TKR, and 633 revision THR were included. Expected length of stay fell from 16.0 days (95% CI: 14.9 to 17.2) in 1997 to 5.4 (5.2 to 5.6) in 2014 for primary TKR, and from 14.4 (13.7 to 15.0) to 5.6 (5.4 to 5.8) for primary THR leading to savings of £1,537 and £1,412, respectively. Length of stay fell from 29.8 (17.5 to 50.5) to 11.0 (8.3 to 14.6) for revision TKR, and from 18.3 (11.6 to 28.9) to 12.5 (9.3 to 16.8) for revision THR, but no significant reduction in reimbursement was estimated. The estimated effect of year of surgery remained similar when patient characteristics were included.

Conclusions

Length of stay for joint replacement fell substantially from 1997 to 2014. These reductions have translated into substantial savings. While patient characteristics affect length of stay

For peer review only

and reimbursement, patient profiles have remained broadly stable over time. The observed reductions appear to be mostly explained by improved efficiency.

ARTICLE SUMMARY

Strengths and limitations of this study

- Routinely collected data provided real-world information on trends in length of stay following primary knee and hip replacement and revision procedures.
- Patient characteristics were controlled for to assess whether trends in length of stay and associated hospital reimbursement were explained by changes in patient characteristics or improved efficiency.
- Codes used to identify diagnoses of osteoarthritis have not been fully evaluated.

Word count: 3,530

For peer review only

INTRODUCTION

Knee and hip replacements relieve pain and improve function for individuals with end-stage arthritis, leading to substantial gains in an individual's quality of life.[1,2] The procedures are a cost-effective use of healthcare resources, with their costs justified by the expected health gains for the individuals receiving them.[1,2] Rates of joint replacement grew substantially between 1991 and 2006 in the United Kingdom.[3] Demand is projected to continue to rise in the United States up to 2030 and, in turn, the number of revision procedures is also expected to increase.[4]

Satisfying rising demand will require either additional resources, which is unlikely given current funding constraints, or reducing the cost of the procedures. Length of stay is a key component of the overall cost of joint replacement. Greater efficiency should reduce the expected length of stay for an individual requiring surgery and lead to a cost-saving, all else being equal. If patient health outcomes are not compromised in the process, greater efficiency would make joint replacements a more cost-effective procedure.

Length of stay following joint replacement has fallen substantially.[5–11] Although such reductions may be explained by improved efficiency, they may also be explained by changes in patient profiles over time. As patient characteristics are likely to influence length of stay, changes in these characteristics would partially explain the observed changes in stay length. Individuals at greater risk of poor health outcomes, due to either pre-operative comorbidities or post-operative complications, can be expected to have longer stays. Social determinants can also be expected to influence length of stay.

We assessed the extent to which length of stay following joint replacement decreased in the English National Health Service from 1997 to 2014 and whether such changes have been translated into reduced reimbursement to hospitals, all else being equal. We considered the degree to which observed changes can be explained by improved efficiency or changes in patient profiles.

METHODS

Study Design

Individuals with a diagnosis of osteoarthritis (OA) or rheumatoid arthritis (RA) were identified from primary care records. Linked hospital records were used to identify the procedures of interest following diagnosis: primary total knee replacement (TKR), primary

For peer review only

total hip replacement (THR), revision TKR, and revision THR. Length of stay and hospital reimbursement for these surgeries were calculated. Trends in length of stay over time were assessed using univariable generalised linear models. How such reductions have translated into lower hospital reimbursement, all else being equal, was assessed by examining trends in reimbursement, at current rates, in a similar manner. Patient characteristics were added as explanatory variables to the models to consider whether trends were explained by changes in efficiency or changes in patient profiles.

Setting

Primary care records were derived from the Clinical Practice Research Datalink (CPRD). CPRD contains medical records and demographic details for around 7% of the UK population, with those included representative of the general population.[12] A subset of practices within CPRD were linked to inpatient hospital records, provided by Hospital Episode Statistics Admitted Patient Care (HES APC). HES APC contains data on all NHS hospital admissions in England, with each uninterrupted inpatient stay at one hospital recorded as a spell (the time a patient spent in a particular hospital).[13] The CPRD extract used to identify diagnoses began in January 1995. The HES APC extract used to identify events of interest began in April 1997. Linked data from both databases were extracted up to March 2014.

Participants

We extracted CPRD records for individuals with a diagnosis of either OA or RA. Study participants were identified separately for procedures relating to the knee and hip. For procedures relating to the knee, CPRD records were extracted for those individuals with a diagnosis recorded for RA or knee OA. For procedures relating to the hip, records were extracted for individuals with a diagnosis of RA or hip OA.

Some individuals had diagnostic codes for both RA and OA. It was not possible to determine which of their diagnoses drove the decision for joint replacement. The need a joint replacement for those with RA is often dependent on the development of secondary OA. Diagnosis of RA was therefore taken as the index diagnosis from which follow-up started when patients had both diagnoses recorded.

CPRD records were then linked with HES APC data and procedures of interest after the index diagnosis were identified. Only the first instance of each procedure for an individual was

For peer review only

included in the analysis, and any bilateral procedures were excluded. Although no individuals contributed multiple records of the same surgery, an individual could provide data on multiple procedures.

Variables

Diagnoses of RA, knee OA, and hip OA were identified using clinical (READ) codes within CPRD. Events of interest (TKR, TKR revision, THR, and THR revision) were identified using intervention (OPCS) codes in HES APC.

The date of a procedure recorded in HES APC was used to identify the year of surgery and, given an individual's year of birth recorded in CPRD, age at surgery. Gender was recorded in CPRD. Diagnosis codes in HES APC were used to identify diseases included in the Royal College of Surgeons (RCS) Charlson score, a measure of comorbidities, and calculate the overall summary score (0, 1, 2 or 3+).[14] Index of Multiple Deprivation (IMD) quintiles, a measure of socio-economic status (with IMD quintile 5 implying the highest level of deprivation), were derived from CPRD.

Length of stay was recorded within HES APC by days for the spell in which a procedure occurred. Hospital spells were assigned to Healthcare Resource Groups (HRGs), which group clinically similar treatments using common levels of healthcare resources. The 2017/18 draft prices were used to estimate hospital reimbursement of spells based on the HRG, the method of admission (elective or non-elective), the expected physiotherapy cost, and any stay longer than the trim-point, which is the upper limit of length of stay incorporated in the HRG cost. While variation between individuals' hospital reimbursement were influenced by HRG codes and type of admission, high estimates were driven primarily by stays longer than the trim-point. Changes in reimbursement over time, therefore, reflect the effect of changing length of stay on reimbursement to hospitals from the NHS at current levels, not the accounting costs incurred by hospitals or historical reimbursement rates. Secondary diagnosis codes that were ineligible for grouping were dropped from the record. Reimbursement was classified as missing if the primary diagnosis or any other variable required for the grouper software was ineligible.

Statistical methods

Trends in length of stay and hospital reimbursement over time for each procedure were assessed using univariable regression models that used the year of surgery as the

For peer review only

explanatory factor. Multivariable regressions that included patient characteristics were also specified. We compared the estimated effect of year of surgery on length of stay and hospital reimbursement with and without patient characteristics to determine the extent to which a trend over time could be explained by change in patient characteristics or improvements in efficiency. A similar approach has previously been used to assess whether variations in length of stay between types of providers are due to differences in efficiency or patient selection.[15]

As both length of stay and hospital reimbursement are non-negative and right-skewed, generalised linear models (GLMs) with a gamma distribution and a log-link were specified.[16] For continuous variables, non-linearity was incorporated using restricted cubic splines. Interactions between year and patient characteristics were tested for in the multivariable model through a comparison, using ANOVA, of models with and without interaction terms. The Bayesian information criterion (BIC) was used to decide whether to specify a variable's relationship as non-linear and, if included, how many knots to include. Compared to the Akaike information criterion (AIC), BIC favours parsimony and has been recommended for explanatory models.[17]

Year of surgery, diagnosis (RA or OA), age, gender, RCS Charlson score, and IMD quintile were included as explanatory variables in each of the multivariable models. The code for RA was omitted from calculations of the RCS Charlson score as RA was specified as a separate variable. The estimated effect of the RCS Charlson score is that of comorbidities aside from RA. The two highest RCS Charlson score categories, 2 and 3+, were combined into the category 2+ as there were few scores of 3 or more.

The only explanatory variable with missing data was the IMD quintile, which was missing for 9 TKR and 7 THR; these individuals were omitted from all regressions. There were no missing data for length of stay. Reimbursement estimates could not be calculated for the 2.3% of spells for which the HRG grouper did not produce a valid HRG code. Spells without HRG codes were dropped from the regressions on hospital reimbursement, but were included in the analysis of length of stay.

Data analysis was primarily undertaken in R 3.3.1,[18] using dplyr[19] for data manipulation, rms[20] for fitting regression models, and ggplot2[21] to produce plots. HRG4+ codes were derived using the 2015/16 Reference Costs Grouper.

For peer review only

RESULTS

Study participants

Records from 21,128 patients with OA or RA were included in the analysis, with 10,260 undergoing primary TKR, 10,961 primary THR, 505 TKR revision, and 633 THR revision in the study period. Inclusion of study participants is described in a flow chart in Figure 1. Around 2% of individuals had diagnoses of both OA and RA. RA was taken as the index diagnosis for these individuals.

Individuals' characteristics at the time of surgery are described in Table 1 and are stratified by year of surgery in the appendix. Age, gender, and socio-economic status, measured by IMD Quintile, remained relatively stable over the study period. While the proportion of patients with a diagnosis of RA fell over time, the number of comorbidities at surgery, as measured by the RCS Charlson score, increased.

Trends in expected length of stay and hospital reimbursement over time

Observed length of stay and hospital reimbursement by year of surgery are detailed in the appendix. The estimated effects of year of surgery on length of stay from univariable regressions are presented in Figure 2. Expected length of stay fell from 16.0 days (95% confidence interval: 14.9 to 17.2) in 1997 to 5.4 (5.2 to 5.6) in 2014 for primary TKR, from 14.4 (13.7 to 15.0) to 5.6 (5.4 to 5.8) for primary THR, from 29.8 (17.5 to 50.5) to 11.0 (8.3 to 14.6) for revision TKR, and from 18.3 (11.6 to 28.9) to 12.5 (9.3 to 16.8) for revision THR. Including patient characteristics in the regression models had little effect on the association between year of surgery and length of stay and hospital reimbursement, as shown in Table 2. For example, multivariable adjustment for patient characteristics changed the expected reduction in length of stay for primary THR from 5% (RE: 0.95 [95% CI: 0.94 to 0.95]) to 6% per year (RE: 0.94 [95% CI: 0.94 to 0.95]).

Estimated trends in hospital reimbursement from univariable regressions were broadly similar to those estimated for length of stay (Figure 3). Expected mean hospital reimbursement fell from £7,634 (£7,464 to £7,808) in 1997 to £6,097 (£6,016 to £6,179) in 2014 for primary TKR, from £7,300 (£7,187 to £7,414) to £5,888 (£5,827 to £5,949) for primary THR, from £9,088 (£7,727 to £10,689) to £7,296 (£6,717 to £7,926) for revision TKR, and from £7,655 (£6,385 to £9,177) to £7,564 (£6,743 to £8,485) for revision THR. Including patient characteristics in the regression models did not remove the estimated association

For peer review only

between year of surgery and hospital reimbursement, as shown in Table 3. For example, the expected reduction in length of stay for revision TKR was 1% per year (RE: 0. 99 [95% CI: 0.97 to 1.00]) in the unadjusted model and 1% per year (RE: 0. 99 [95% CI: 0.98 to 1.00]) in the multivariable model.

Impact of patient characteristics on expected length of stay and hospital reimbursement

Tables 2 and 3 detail the relative effects of patient characteristics on length of stay and hospital reimbursement from the fully specified models. No interactions between year of surgery and patient characteristics were identified. Higher age at surgery was associated with longer stays for each procedure. Where the effect of age was non-linear, its impact was more pronounced among older patients. Holding all other explanatory variables at their average, expected length of stay increased from 5.7 (5.5 to 5.9) for a 55-year-old to 9.1 (8.7 to 9.5) for an 85-year-old undergoing primary TKR, from 6.2 (5.9 to 6.4) to 10.6 (10.1 to 11.0) for primary THR, from 9.6 (6.5 to 14.1) to 14.4 (9.5 to 21.8) for revision TKR, and from 12.4 (9.4 to 16.3) to 27.3 (21.0 to 35.5) for revision THR. Similar upward trends were estimated for hospital reimbursement for each of the procedures of interest. With other explanatory variables held at their average value, expected reimbursement increased from £5,856 (£5,791 to £5,923) for a 55-year-old to £6,553 (£6,471 to £6,636) for an 85-year-old undergoing primary TKR, from £5,792 (£5,734 to £5,850) to £6,596 (£6,520 to £6,672) for primary THR, from £6,409 (£5,811 to £7,069) to £7,691 (£6,976 to £8,480) for revision TKR, and from £8,071 (£7,292 to £8,934) to £10,269 (£9,315 to £11,320) for revision THR. Plots of the partial effect of age on length of stay and hospital reimbursement are provided in the appendix.

DISCUSSION

Principal findings

Length of stay for primary TKR and THR fell significantly in the English NHS between 1997 and 2014. Expected length of stay fell by 11 days for primary TKR and 9 days for primary THR. The trajectory of the fall in expected length of stay was estimated as linear for primary THR, but had slowed somewhat towards the end of the study period for primary TKR. Length of stay also fell significantly for revision TKR, by 19 days between 1997 and 2014. Although a drop of 6 days was estimated for revision THR, this fall was not statistically significant.

For peer review only

Reductions is length of stay have, all else being equal, led to significant reductions in hospital reimbursement, which reflect costs from the NHS perspective. Reimbursement was reduced by £1,537 for primary TKR and £1,412 for primary THR than otherwise would have been the case. The trajectories of these reductions in reimbursement were broadly in line with those of length of stay. The estimated reductions in reimbursement due to reduced length of stay for the revision procedures, £1,792 for revision TKR and £91 for revision THR, were not statistically significant.

Age at surgery was associated with increased length of stay and reimbursement for each procedure with, in general, greater increases among older patients. RA and other comorbidities were associated with significantly higher length of stay and hospital reimbursement. Lower socioeconomic status was associated with a significant increase in length of stay and reimbursement for primary procedures. Male gender was associated with significantly shorter stays for primary procedures.

While the age, gender, and socioeconomic status of those receiving joint replacement and revision surgery remained stable over time, individuals undergoing surgery had more comorbidities in more recent years although the proportion diagnosed with RA fell. Controlling for patient characteristics had little effect on the association between year of surgery and length of stay or associated hospital reimbursement. These results imply that the downward trends in length of stay and associated hospital reimbursement are generally explained by increased efficiency.

Study findings in context

Previous studies have also found length of stay to have fallen over recent decades following TKR and THR.[5–11] The downward trends in length of stay appear to be primarily due to increased efficiency, rather than changes in patient characteristics.

A key driver of the efficiency gains in joint replacement has likely been a move towards ‘fast-track’ arthroplasty.[22] Enhanced recovery programmes (ERPs) have become increasingly prominent in orthopaedic surgery, aiming to standardise routine perioperative care, reduce length of hospital stay, and promote rapid recovery.[23] These programmes have been found to reduce length of stay for a range of surgical procedures,[24] including knee and hip replacement.[25] The implementation of ERPs has been associated with either equivalent[23,26] or improved health outcomes[25], and there is little evidence that ERPs lead to an increase in readmission rates.[24,27]

For peer review only

While ERPs have gained prominence, there remains scope for further implementation. As demonstrated in our study both older age at surgery and comorbidities are associated with higher length of stay following TKR and THR. ERPs have not only been demonstrated to be appropriate for such patients, but the reductions in length of stay are, in fact, greater than for younger patients with fewer comorbidities.[28] Greater implementation of ERPs among such groups can, then, be expected to lead to further reductions in length of stay following joint replacement and additional cost-savings from the NHS perspective.

In the UK, government policy has also encouraged the use of specialist centres for joint replacement, and this has likely also contributed to the observed reductions in length of stay. Patients in specialised centres typically have lower length of stay.[9] This difference is not only due to patient selection as even after controlling for differences in the characteristics of those undergoing surgery in different centres, length of stay following THR is lower when surgery is undertaken at a specialist treatment centre.[15]

As would be expected, the reductions in length of stay translate into cost-savings for the NHS with reduced reimbursement to hospitals than otherwise would be the case. This has also been seen in the US where, although the overall costs for joint replacement have risen, falling length of stay has attenuated the increase.[29,30]

As observed here, the age and gender of those undergoing surgery has been relatively stable between 1991 and 2006.[3] Older age at surgery, particularly those over 80[6,31], and of lower income[31] are both associated with longer stays. While gender has typically not been found to have an effect on length of stay[6] or costs[32], being female was associated with longer stays following TKR in one study.[31] Over the study period individuals appear to have an increasing number of comorbidities at the time of surgery for all of the procedures considered. This trend has previously been reported in the US.[5,7,30] Comorbidities have been associated with longer hospital stays and greater costs following primary joint replacement.[31,32] The proportion of those undergoing primary TKR with a diagnosis RA though fell over time in this study. This trend has also previously been reported and is likely explained by therapeutic advances, such as the introduction of biologic therapies.[33] The relative impact of RA on length of stay and costs is not well known, but the increases estimated here may be explained by greater morbidity before surgery and the use of medications such as steroids and immunosuppressants.

For peer review only

Strength and limitations of this study

Routinely collected data is not collected primarily to inform research, leading to a number of possible limitations. Coding accuracy is of particular concern. While the diagnosis of RA[34] and the coding of primary TKR and primary THR[35] have previously been validated in CPRD and HES APC respectively, OA coding has not been thoroughly evaluated. If those misclassified as not having an OA diagnosis differed systematically from those with a recorded OA diagnosis, the external validity of our results may be affected.

A number of patient characteristics were controlled for to assess whether trends in length of stay and reimbursement were explained by changes in patient characteristics or improved efficiency. These included age, gender, comorbidities and socioeconomic status. However, other patient characteristics may have changed over time and influenced length of stay or reimbursement. Moreover, the changes in reported comorbidities may reflect changes in data capture over time.

A further limitation of this study is that we only examined the length of stay and hospital reimbursement of the spell for each individual's first recorded primary and revision procedure. This may have led to an underestimation of the total reimbursement to hospitals. In particular, revision procedures can have multiple stages and subsequent revision procedures may be required, which may lead to a higher overall hospital reimbursement than reported here. Furthermore, readmissions have not been included in the study. If reduced length of stay has been accompanied by an increase in the number of readmissions, then the reductions in resource use will have been overstated.

The primary strength of this study is that, despite potential concerns around coding accuracy, it was informed by routinely collected data which provided real-world evidence of trends in length of stay. Sample sizes were large, particularly for primary TKR and primary THR, allowing a thorough analysis of trends over time, and the data were representative of the experience of the NHS in England.

The costs estimated here reflect reimbursement to hospitals by the NHS. This reflects costs from the NHS perspective and helps to inform decision-making for the health system as a whole. By using current reimbursement rates it has been possible to consider how changes in length of stay have translated into changes in reimbursement, all else being equal. However, trends in overall reimbursement likely follow a different trend given changes in other factors, such as the prices of implants. In addition, the accounting costs incurred by

For peer review only

hospitals likely differ to the reimbursement received and have not been assessed in this study. Trends in the costs incurred by hospitals may differ, for example, if they are less sensitive to changes in length of stay.

Policy implications and areas for future research

Expected length of stay associated with TKR and THR fell significantly between 1997 and 2014. This appears to be due to improved efficiency rather than changes in patient profiles. The reductions in length of stay have led, all else being equal, to cost-savings from a NHS perspective. If health outcomes have not worsened, reductions in length of stay will have improved the cost-effectiveness of joint replacement relative to what otherwise would have been the case.

With stretched healthcare budgets, further improvements in efficiency will likely be necessary for supply to keep pace with the rising demand for joint replacement procedures. Additional research into the drivers of the efficiency gains identified in this study would help inform future policy decisions.

For peer review only

ACKNOWLEDGEMENTS

The authors would like to thank Miss Susan Thwaite (National Rheumatoid Arthritis Society) for her role as the patient and public representative and her role in the study steering committee. We also thank Dr Jennifer A. de Beyer of the Centre for Statistics in Medicine, University of Oxford, for English language editing.

FUNDING

DPA is funded by a National Institute for Health Research Clinician Scientist award (CS-2013-13-012). This article presents independent research funded by the National Institute for Health Research (NIHR). The views expressed are those of the authors and not necessarily those of the NHS, the NIHR or the Department of Health. This work was supported by the NIHR Biomedical Research Centre, Oxford.

CONTRIBUTORS

EB, CE, DWM, AS, CC, NKA, RPV and DPA all made substantial contributions to conception and design of the study. EB, RPV and DPA undertook the statistical analysis. EB, RPV and DPA drafted the manuscript with CE, DWM, AS, CC, and NKA revising it for important intellectual content. All authors read and approved the final manuscript.

COMPETING INTERESTS

All authors have completed the ICMJE uniform disclosure form at www.icmje.org/coi_disclosure.pdf and declare: NKA has received personal fees from Freshfields Bruckhaus Deringer, Bioventus, Flexion, Merck, and Regeneron, all outside the submitted work. DPA reports grants from Amgen, Servier and UCB Biopharma, and non-financial support from Amgen, all outside the submitted work. DWM reports grants and personal fees from Zimmer Biomet. In addition, DWM has various patents related to Unicompartmental Knee Replacement (Zimmer Biomet) with royalties paid.

ETHICAL APPROVAL

Approval for the study was granted by the CPRD Independent Scientific Advisory Committee..

DATA SHARING

CPRD data with HES linkage were provided under a licence that does not permit sharing. Data are obtainable from CPRD subject to a full application.

For peer review only

REFERENCES

1 Pivec R, Johnson AJ, Mears SC, *et al.* Hip arthroplasty. *Lancet* 2012;**380**:1768–77. doi:10.1016/S0140-6736(12)60607-2

2 Carr AJ, Robertsson O, Graves S, *et al.* Knee replacement. *Lancet* 2012;**379**:1331–40. doi:10.1016/s0140-6736(11)60752-6

3 Culliford DJ, Maskell J, Beard DJ, *et al.* Temporal trends in hip and knee replacement in the United Kingdom: 1991 TO 2006. *J Bone Jt Surg - Br Vol* 2010;**92–B**:130–5. doi:10.1302/0301-620X.92B1.22654

4 Kurtz S, Ong K, Lau E, *et al.* Projections of primary and revision hip and knee arthroplasty in the United States from 2005 to 2030. *J Bone Joint Surg Am* 2007;**89**:780–5. doi:10.2106/JBJS.F.00222

5 Cram P, Lu X, Kates SL, *et al.* Total Knee Arthroplasty Volume, Utilization, and Outcomes Among Medicare Beneficiaries, 1991-2010. *JAMA* 2012;**308**:1227. doi:10.1001/2012.jama.11153

6 Smith IDM, Elton R, Ballantyne JA, *et al.* Pre-operative predictors of the length of hospital stay in total knee replacement. *J Bone Jt Surg - Br Vol* 2008;**90–B**:1435–40. doi:10.1302/0301-620X.90B11.20687

7 Cram P. Clinical Characteristics and Outcomes of Medicare Patients Undergoing Total Hip Arthroplasty, 1991-2008. *Jama* 2011;**305**:1560. doi:10.1001/jama.2011.478

8 Cookson R, Laudicella M. Do the poor cost much more? The relationship between small area income deprivation and length of stay for elective hip replacement in the English NHS from 2001 to 2008. *Soc Sci Med* 2011;**72**:173–84. doi:10.1016/j.socscimed.2010.11.001

9 Husted H, Jensen CM, Solgaard S, *et al.* Reduced length of stay following hip and knee arthroplasty in Denmark 2000-2009: From research to implementation. *Arch Orthop Trauma Surg* 2012;**132**:101–4. doi:10.1007/s00402-011-1396-0

10 Jimenez García R, Villanueva Martínez M, Fernández de Las-Penas C, Hernandez Barrera V, Rios Luna A, Carrasco Garrido P, Lopez de Andres A, Jimenez Trujillo I, San

For peer review only

Roman Montero J, Gil de Miguel A. Trends in primary total hip arthroplasty in Spain from 2001 to 2008: Evaluating changes in demographics, comorbidity, incidence rates, length of stay, costs and mortality. *BMC Musculoskelet Disord* 2011;**12**:43. doi:10.1186/1471-2474-12-43

11 Pamilo KJ, Peltola M, Mäkelä K, *et al.* Is hospital volume associated with length of stay, re-admissions and reoperations for total hip replacement? A population-based register analysis of 78 hospitals and 54,505 replacements. *Arch Orthop Trauma Surg* 2013;**133**:1747–55. doi:10.1007/s00402-013-1860-0

12 Herrett E, Gallagher AM, Bhaskaran K, *et al.* Data Resource Profile: Clinical Practice Research Datalink (CPRD). *Int J Epidemiol* 2015;**44**:827–36. doi:10.1093/ije/dyv098

13 Herbert A, Wijlaars L, Zylbersztejn A, *et al.* Data Resource Profile: Hospital Episode Statistics Admitted Patient Care (HES APC). *Int J Epidemiol* 2017;:1–10. doi:10.1093/ije/dyx015

14 Armitage JN, Van Der Meulen JH. Identifying co-morbidity in surgical patients using administrative data with the Royal College of Surgeons Charlson Score. *Br J Surg* 2010;**97**:772–81. doi:10.1002/bjs.6930

15 Siciliani L, Sivey P, Street A. DIFFERENCES IN LENGTH OF STAY FOR HIP REPLACEMENT BETWEEN PUBLIC HOSPITALS, SPECIALISED TREATMENT CENTRES AND PRIVATE PROVIDERS: SELECTION OR EFFICIENCY? *Health Econ* 2013;**22**:234–42. doi:10.1002/hec.1826

16 Mihaylova B, Briggs A, O'Hagan A, *et al.* Review of statistical methods for analysing healthcare resources and costs. *Health Econ* 2011;**20**:897–916. doi:10.1002/hec.1653

17 Aho K, Derryberry D, Peterson T. Model selection for ecologists: the worldview of AIC and BIC. *Ecology* 2014;**95**:631–6. doi:10.1890/13-1452.1

18 R Core Team. R: A language and environment for statistical computing. R Foundation for Statistical Computing, Vienna, Austria. 2016.

19 Wickham H, Francois R. dplyr: A Grammar of Data Manipulation. R package version 0.5.0. 2016.

For peer review only

20 Harrell , FE. rms: Regression Modeling Strategies. R package version 5.1-0. 2017.

21 Wickham H. *ggplot2: Elegant Graphics for Data Analysis*. New York: : Springer-Verlag 2009. doi:10.1007/978-0-387-98141-3

22 Kehlet H. Fast-track hip and knee arthroplasty. *Lancet* 2013;**381**:1600–2. doi:10.1016/S0140-6736(13)61003-X

23 Maempel JF, Walmsley PJ. Enhanced recovery programmes can reduce length of stay after total knee replacement without sacrificing functional outcome at one year. *Ann R Coll Surg Engl* 2015;**97**:563–7. doi:10.1308/rcsann.2015.0016

24 Paton F, Chambers D, Wilson P, *et al.* Effectiveness and implementation of enhanced recovery after surgery programmes: a rapid evidence synthesis. *BMJ Open* 2014;**4**:e005015–e005015. doi:10.1136/bmjopen-2014-005015

25 Khan SK, Malviya A, Muller SD, *et al.* Reduced short-term complications and mortality following Enhanced Recovery primary hip and knee arthroplasty: results from 6,000 consecutive procedures. *Acta Orthop* 2014;**85**:26–31. doi:10.3109/17453674.2013.874925

26 Jones EL, Wainwright TW, Foster JD, *et al.* A systematic review of patient reported outcomes and patient experience in enhanced recovery after orthopaedic surgery. *Ann R Coll Surg Engl* 2014;**96**:89–94. doi:10.1308/003588414X13824511649571

27 Husted H, Otte KS, Kristensen BB, *et al.* Readmissions after fast-track hip and knee arthroplasty. *Arch Orthop Trauma Surg* 2010;**130**:1185–91. doi:10.1007/s00402-010-1131-2

28 Starks I, Wainwright TW, Lewis J, *et al.* Older patients have the most to gain from orthopaedic enhanced recovery programmes. *Age Ageing* 2014;**43**:642–8. doi:10.1093/ageing/afu014

29 Cary MP, Baernholdt M, Merwin EI. Changes in Payment Regulation and Acute Care Use for Total Hip Replacement: Trends in Length of Stay, Costs, and Discharge, 1997-2012. *Rehabil Nurs* 2016;**41**:67–77. doi:10.1002/rnj.210

30 Molloy IB, Martin BI, Moschetti WE, *et al.* Effects of the Length of Stay on the Cost of

For peer review only

Total Knee and Total Hip Arthroplasty from 2002 to 2013. *J Bone Jt Surg* 2017;**99**:402–7. doi:10.2106/JBJS.16.00019

31 El Bitar YF, Illingworth KD, Scaife SL, *et al.* Hospital Length of Stay following Primary Total Knee Arthroplasty: Data from the Nationwide Inpatient Sample Database. *J Arthroplasty* 2015;**30**:1710–5. doi:10.1016/j.arth.2015.05.003

32 Shah AN, Vail TP, Taylor D, *et al.* Comorbid illness affects hospital costs related to hip arthroplasty: Quantification of health status and implications for fair reimbursement and surgeon comparisons. *J Arthroplasty* 2004;**19**:700–5. doi:10.1016/j.arth.2004.02.034

33 Harty L, O'Toole G, FitzGerald O. Profound reduction in hospital admissions and musculoskeletal surgical procedures for rheumatoid arthritis with concurrent changes in clinical practice (1995-2010). *Rheumatology (Oxford)* 2015;**54**:666–71. doi:10.1093/rheumatology/keu340

34 Thomas SL, Edwards CJ, Smeeth L, *et al.* How accurate are diagnoses for rheumatoid arthritis and juvenile idiopathic arthritis in the general practice research database? *Arthritis Care Res* 2008;**59**:1314–21. doi:10.1002/art.24015

35 Hawley S, Delmestri A, Judge A, *et al.* Total Hip and Knee Replacement Among Incident Osteoarthritis and Rheumatoid Arthritis Patients Within the UK Clinical Practice Research Datalink (CPRD) Compared to Hospital Episode Statistics (HES): A Validation Study. *Pharmacoepidemiol Drug Saf* 2016.

For peer review only

Table 1. Patient characteristics by procedure

	Primary TKR	Primary TKR	Revision TKR	Revision THR
Number of study participants	10,260	10,961	505	633
Age (mean (sd))	70.01 (9.24)	68.93 (10.53)	69.43 (9.93)	70.34 (10.91)
Gender: male (n (%))	4426 (43.1)	4525 (41.3)	233 (46.1)	264 (41.7)
Year of surgery (median [IQR])	2009 [2006, 2011]	2008 [2005, 2011]	2009 [2007, 2011]	2009 [2006, 2011]
Diagnoses recorded in CPRD				
Rheumatoid arthritis (n (%))	851 (8.3)	639 (5.8)	65 (12.9)	92 (14.5)
Diagnoses recorded in HES APC				
Myocardial infarction (n (%))	138 (1.3)	140 (1.3)	6 (1.2)	14 (2.2)
Congestive cardiac failure (n (%))	96 (0.9)	137 (1.2)	8 (1.6)	14 (2.2)
Peripheral vascular disease (n (%))	101 (1.0)	102 (0.9)	10 (2.0)	8 (1.3)
Cerebrovascular disease (n (%))	54 (0.5)	56 (0.5)	3 (0.6)	5 (0.8)
Dementia (n (%))	25 (0.2)	37 (0.3)	0 (0.0)	10 (1.6)
Chronic pulmonary disease (n (%))	1091 (10.6)	998 (9.1)	58 (11.5)	55 (8.7)
Rheumatological disease (n (%))	593 (5.8)	474 (4.3)	38 (7.5)	50 (7.9)
Liver disease (n (%))	20 (0.2)	22 (0.2)	0 (0.0)	5 (0.8)
Diabetes mellitus (n (%))	991 (9.7)	755 (6.9)	56 (11.1)	45 (7.1)
Hemiplegia or paraplegia (n (%))	18 (0.2)	17 (0.2)	4 (0.8)	5 (0.8)
Renal disease (n (%))	202 (2.0)	235 (2.1)	11 (2.2)	20 (3.2)
Any malignancy (n (%))	78 (0.8)	97 (0.9)	7 (1.4)	9 (1.4)
Metastatic solid tumour (n (%))	6 (0.1)	10 (0.1)	1 (0.2)	2 (0.3)
AIDS HIV infection (n (%))	0 (0.0)	1 (0.0)	0 (0.0)	0 (0.0)
RCS Charlson score (n (%))				
0	7443 (72.5)	8413 (76.8)	342 (67.7)	455 (71.9)
1	2302 (22.4)	2110 (19.3)	130 (25.7)	124 (19.6)
2	437 (4.3)	353 (3.2)	29 (5.7)	45 (7.1)
3+	78 (0.8)	85 (0.8)	4 (0.8)	9 (1.4)

For peer review only

IMD quintiles (n (%))				
1	2330 (22.7)	2779 (25.4)	128 (25.3)	208 (32.9)
2	2487 (24.2)	2794 (25.5)	120 (23.8)	155 (24.5)
3	2383 (23.2)	2479 (22.6)	117 (23.2)	127 (20.1)
4	1930 (18.8)	1923 (17.5)	105 (20.8)	84 (13.3)
5	1121 (10.9)	980 (8.9)	35 (6.9)	59 (9.3)
Missing	9 (0.1)	6 (0.1)	0 (0.0)	0 (0.0)
Ethnicity (n (%))				
Non-White	240 (2.3)	60 (0.5)	15 (3.0)	6 (0.9)
White	9073 (88.4)	9643 (88.0)	460 (91.1)	575 (90.8)
Missing	947 (9.2)	1258 (11.5)	30 (5.9)	52 (8.2)

For peer review only

Table 2. Multivariable regressions for length of stay

	Primary TKR (RE (95% CI))		Primary THR (RE (95% CI))		Revision TKR (RE (95% CI))		Revision THR (RE (95% CI))	
	Unadjusted	Adjusted	Unadjusted	Adjusted	Unadjusted	Adjusted	Unadjusted	Adjusted
Year of surgery	0.93 (0.92 to 0.94)	0.93 (0.93 to 0.94)	0.95 (0.94 to 0.95)	0.94 (0.94 to 0.95)	0.94 (0.90 to 0.99)	0.96 (0.92 to 1.00)	1.02 (0.96 to 1.07)	1.02 (0.96 to 1.07)
Year of surgery†	1.01 (1.00 to 1.02)	1.01 (1.00 to 1.02)					0.93 (0.87 to 1.00)	0.91 (0.85 to 0.98)
Age		1.00 (1.00 to 1.00)		1.00 (1.00 to 1.01)		0.99 (0.96 to 1.02)		1.03 (1.02 to 1.04)
Age†		1.02 (1.01 to 1.02)		1.02 (1.01 to 1.02)		1.03 (0.99 to 1.07)		
Gender: Male		0.98 (0.95 to 1.00)		0.94 (0.91 to 0.96)		1.03 (0.78 to 1.37)		0.89 (0.72 to 1.09)
Diagnosis: RA		1.17 (1.11 to 1.22)		1.17 (1.10 to 1.25)		2.35 (1.56 to 3.68)		1.35 (1.02 to 1.81)
RCS Charlson score*								
0		*Ref*		*Ref*		*Ref*		*Ref*
1		1.17 (1.13 to 1.21)		1.13 (1.09 to 1.18)		0.89 (0.64 to 1.25)		1.27 (0.98 to 1.67)
2+		1.35 (1.26 to 1.44)		1.43 (1.32 to 1.55)		1.49 (0.79 to 3.19)		1.95 (1.31 to 3.03)
IMD quintiles								
1		*Ref*		*Ref*		*Ref*		*Ref*
2		1.04 (1.01 to 1.08)		1.01 (0.97 to 1.05)		1.45 (0.98 to 2.15)		0.92 (0.70 to 1.20)
3		1.07 (1.04 to 1.11)		1.02 (0.97 to 1.06)		1.19 (0.80 to 1.78)		0.85 (0.64 to 1.12)

For peer review only

4	1.07 (1.03 to 1.11)	1.05 (1.00 to 1.10)	1.34 (0.90 to 2.02)	0.90 (0.65 to 1.25)
5	1.11 (1.06 to 1.16)	1.12 (1.06 to 1.18)	0.94 (0.53 to 1.79)	0.99 (0.70 to 1.45)

Explanatory factors' relative effect (RE) on days of length of stay with 95% confidence interval (CI) from univariable and multivariable regressions. [†]indicates knots for restricted cubic spline. *RA codes were omitted from the calculation of the RCS Charlson score and groups 2 and 3+ combined into 2+.

For peer review only

Table 3. Multivariable regressions for hospital reimbursement

	Primary TKR (RE (95% CI))		Primary THR (RE (95% CI))		Revision TKR (RE (95% CI))		Revision THR (RE (95% CI))	
	Unadjusted	Adjusted	Unadjusted	Adjusted	Unadjusted	Adjusted	Unadjusted	Adjusted
Year of surgery	0.98 (0.97 to 0.98)	0.98 (0.97 to 0.98)	0.98 (0.98 to 0.98)	0.98 (0.98 to 0.98)	0.99 (0.97 to 1.00)	0.99 (0.98 to 1.00)	1.02 (0.99 to 1.04)	1.02 (1.00 to 1.04)
Year of surgery†	1.02 (1.01 to 1.03)	1.02 (1.01 to 1.03)	1.01 (1.01 to 1.01)	1.01 (1.01 to 1.01)			0.97 (0.95 to 1.00)	0.97 (0.94 to 0.99)
Year of surgery††	0.95 (0.91 to 0.99)	0.94 (0.91 to 0.98)						
Age		1.00 (1.00 to 1.00)		1.00 (1.00 to 1.00)		1.01 (1.00 to 1.01)		1.01 (1.00 to 1.01)
Age†		1.00 (1.00 to 1.00)		1.00 (1.00 to 1.01)				
Age††		1.02 (1.00 to 1.03)						
Gender: Male		1.01 (1.00 to 1.01)		0.99 (0.98 to 1.00)		0.99 (0.92 to 1.07)		0.93 (0.86 to 1.00)
Diagnosis: RA		1.04 (1.03 to 1.05)		1.03 (1.01 to 1.04)		1.37 (1.23 to 1.53)		1.13 (1.02 to 1.26)
RCS Charlson score*								
0		*Ref*		*Ref*		*Ref*		*Ref*
1		1.05 (1.05 to 1.06)		1.03 (1.02 to 1.04)		1.04 (0.96 to 1.14)		1.10 (1.00 to 1.21)
2+		1.13 (1.11 to 1.15)		1.10 (1.08 to 1.12)		1.31 (1.10 to 1.58)		1.13 (0.97 to 1.32)
IMD quintiles								
1		*Ref*		*Ref*		*Ref*		*Ref*
2		1.02 (1.01 to 1.03)		1.00 (0.99 to 1.01)		1.05 (0.95 to 1.16)		0.92 (0.84 to 1.02)

For peer review only

3	1.02 (1.01 to 1.03)	1.00 (0.99 to 1.01)	1.06 (0.96 to 1.18)	0.88 (0.80 to 0.98)
4	1.01 (1.00 to 1.02)	1.01 (1.00 to 1.02)	1.08 (0.97 to 1.20)	0.91 (0.81 to 1.03)
5	1.03 (1.02 to 1.04)	1.02 (1.01 to 1.03)	0.94 (0.81 to 1.10)	0.94 (0.82 to 1.08)

Explanatory factors' relative effect (RE) of diagnosis on hospital reimbursement (at 2016/17 rates in GBP) with 95% confidence interval (CI) from univariable and multivariable regressions. † indicates knots for restricted cubic spline. *RA codes were omitted from the calculation of the RCS Charlson score and groups 2 and 3+ combined into 2+.

For peer review only

Figure 1. Study inclusion flow chart

Figure 2. Trends in length of stay. Estimated effect of year of surgery on length of stay (in days). TKR: total knee replacement, THR: total hip replacement.

Figure 3. Trends in hospital reimbursement. Estimated effect of year of surgery on hospital reimbursement (at 2016/17 rates in GBP). TKR: total knee replacement, THR: total hip replacement.

For peer review only

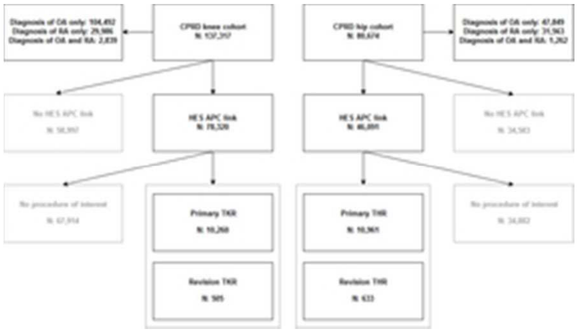

Figure 1. Study inclusion flow chart

24x13mm (300 x 300 DPI)

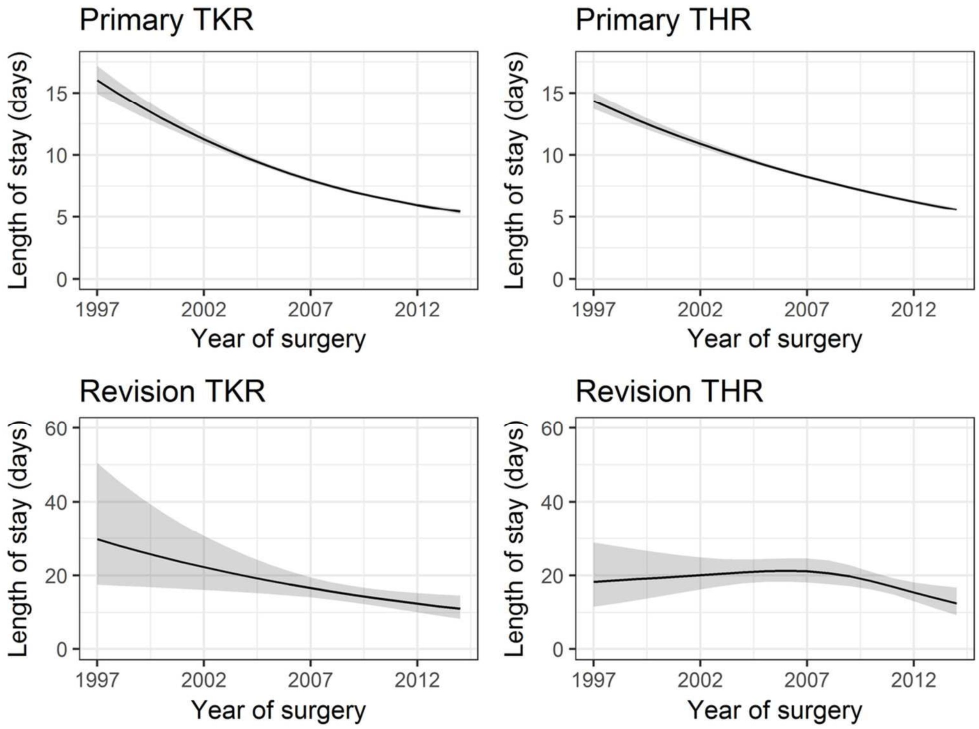

Figure 2. Trends in length of stay. Estimated effect of year of surgery on length of stay (in days). TKR: total knee replacement, THR: total hip replacement.

114x85mm (300 x 300 DPI)

w only

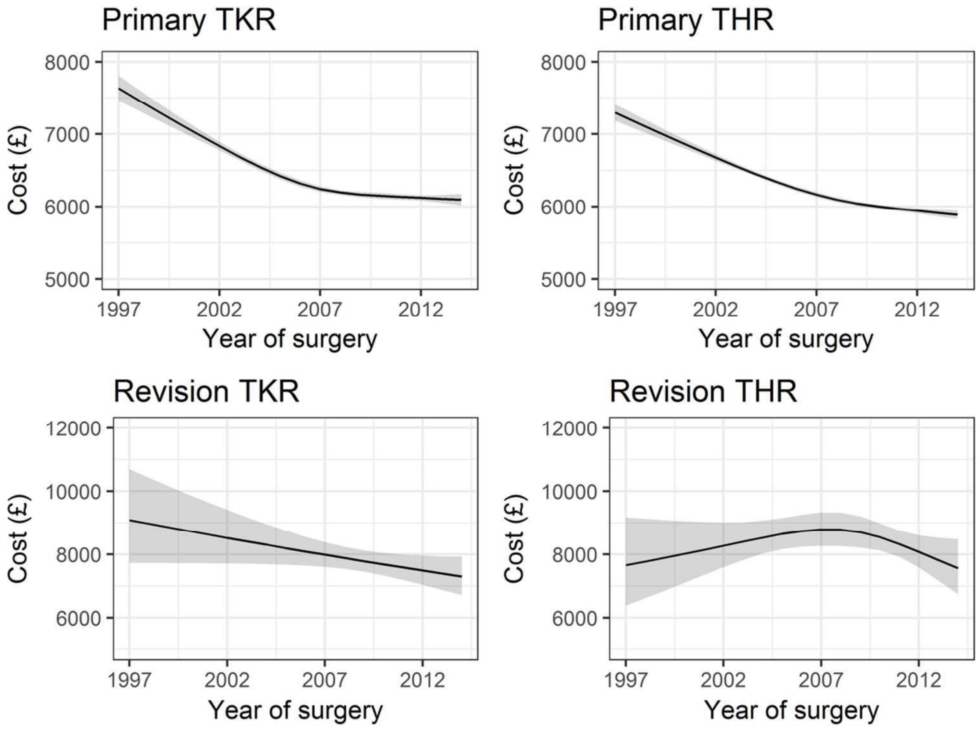

Figure 3. Trends in hospital reimbursement. Estimated effect of year of surgery on hospital reimbursement (at 2016/17 rates in GBP). TKR: total knee replacement, THR: total hip replacement.

114x85mm (300 x 300 DPI)

For peer review only - http://bmjopen.bmj.com/site/about/guidelines.xhtml

APPENDIX

Table of Contents

Patient characteristics by year of surgery 2
Primary total knee replacement 2
Primary total hip replacement 4
Revision total knee replacement 6
Revision total hip replacement 8
Length of stay by year of surgery 10
Hospital costs by year of surgery 12
Partial effect of age on length of stay and costs 14
Partial effect of age at surgery on length of stay from multivariable regressions. Predicted length of stay by age while other explanatory variables are held fixed at their average (median if continuous and mode if categorical). TKR: total knee replacement, THR: total hip replacement. 14
Partial effect of age at surgery on hospital costs (in 2017 GBP) from multivariable regressions. Predicted cost by age while other explanatory variables are held fixed at their average (median if continuous and mode if categorical). TKR: total knee replacement, THR: total hip replacement. 14

For peer review only - http://bmjopen.bmj.com/site/about/guidelines.xhtml

PATIENT CHARACTERISTICS BY YEAR OF SURGERY

Primary total knee replacement

Year of surgery	n	Age (mean (sd))	Gender: Male (n (%))	Rheumatoid arthritis (n (%))	RCS Charlson score (n (%)) 0	1	2	3+	IMD quintiles (n (%)) 1	2	3	4	5
1997	45	72 (9)	18 (40%)	8 (18%)	43 (96%)	2 (4%)	0 (0%)	0 (0%)	7 (16%)	9 (20%)	13 (29%)	8 (18%)	8 (18%)
1998	64	72 (10)	23 (36%)	12 (19%)	57 (89%)	5 (8%)	1 (0%)	1 (0%)	11 (17%)	14 (22%)	16 (25%)	14 (22%)	9 (14%)
1999	114	71 (9)	45 (39%)	6 (5%)	98 (86%)	14 (12%)	2 (0%)	0 (0%)	18 (16%)	32 (28%)	17 (15%)	30 (27%)	16 (14%)
2000	115	72 (9)	55 (48%)	16 (14%)	97 (84%)	16 (14%)	1 (0%)	1 (0%)	22 (19%)	31 (27%)	19 (17%)	21 (18%)	22 (19%)
2001	170	71 (9)	76 (45%)	18 (11%)	152 (89%)	16 (9%)	2 (0%)	0 (0%)	40 (24%)	34 (20%)	43 (25%)	31 (18%)	22 (13%)
2002	268	70 (10)	116 (43%)	28 (10%)	237 (88%)	28 (10%)	3 (0%)	0 (0%)	68 (25%)	67 (25%)	61 (23%)	39 (15%)	33 (12%)
2003	403	71 (9)	173 (43%)	40 (10%)	344 (85%)	51 (13%)	7 (0%)	1 (0%)	95 (24%)	96 (24%)	101 (25%)	64 (16%)	47 (12%)
2004	545	70 (9)	216 (40%)	60 (11%)	458 (84%)	78 (14%)	7 (0%)	2 (0%)	132 (24%)	116 (21%)	146 (27%)	102 (19%)	48 (9%)
2005	605	71 (9)	255 (42%)	60 (10%)	509 (84%)	83 (14%)	11 (0%)	2 (0%)	135 (22%)	129 (21%)	140 (23%)	137 (23%)	62 (10%)
2006	735	71 (9)	315 (43%)	75 (10%)	584 (79%)	135 (18%)	13 (0%)	3 (0%)	157 (21%)	168 (23%)	187 (25%)	142 (19%)	81 (11%)
2007	912	70 (9)	371 (41%)	83 (9%)	742 (81%)	141 (15%)	28 (0%)	1 (0%)	201 (22%)	207 (23%)	204 (22%)	190 (21%)	110 (12%)
2008	981	70 (9)	432 (44%)	73 (7%)	775 (79%)	177 (18%)	28 (0%)	1 (0%)	229 (23%)	243 (25%)	204 (21%)	188 (19%)	115 (12%)

For peer review only - http://bmjopen.bmj.com/site/about/guidelines.xhtml

For peer review only

2009	1045	69 (9)	463 (44%)	71 (7%)	773 (74%)	234 (22%)	29 (0%)	9 (0%)	229 (22%)	282 (27%)	247 (24%)	190 (18%)	96 (9%)
2010	1009	70 (9)	440 (44%)	72 (7%)	744 (74%)	209 (21%)	48 (0%)	8 (0%)	253 (25%)	249 (25%)	230 (23%)	176 (17%)	101 (10%)
2011	1024	69 (9)	461 (45%)	78 (8%)	715 (70%)	249 (24%)	54 (1%)	6 (0%)	229 (22%)	236 (23%)	250 (24%)	200 (20%)	109 (11%)
2012	1018	69 (9)	444 (44%)	69 (7%)	690 (68%)	273 (27%)	48 (0%)	7 (0%)	236 (23%)	269 (26%)	217 (21%)	182 (18%)	113 (11%)
2013	967	70 (9)	416 (43%)	65 (7%)	633 (65%)	272 (28%)	51 (0%)	11 (0%)	216 (22%)	251 (26%)	212 (22%)	184 (19%)	103 (11%)
2014	240	69 (9)	107 (45%)	17 (7%)	153 (64%)	66 (28%)	20 (0%)	1 (0%)	52 (22%)	54 (22%)	76 (32%)	32 (13%)	26 (11%)

For peer review only

Primary total hip replacement

Year of surgery	n	Age (mean (sd))	Gender: Male (n (%))	Rheumatoid arthritis (n (%))	RCS Charlson score (n (%)) 0	1	2	3+	IMD quintiles (n (%)) 1	2	3	4	5
1997	78	69 (10)	32 (41%)	5 (6%)	73 (94%)	5 (6%)	0 (0%)	0 (0%)	21 (27%)	15 (19%)	20 (26%)	16 (21%)	6 (8%)
1998	128	69 (10)	42 (33%)	8 (6%)	120 (94%)	7 (5%)	1 (0%)	0 (0%)	27 (21%)	26 (20%)	34 (27%)	23 (18%)	18 (14%)
1999	195	69 (12)	80 (41%)	11 (6%)	172 (88%)	22 (11%)	1 (0%)	0 (0%)	46 (24%)	47 (24%)	47 (24%)	32 (16%)	23 (12%)
2000	242	70 (10)	104 (43%)	6 (2%)	217 (90%)	22 (9%)	3 (0%)	0 (0%)	57 (24%)	79 (33%)	50 (21%)	37 (15%)	19 (8%)
2001	317	68 (10)	137 (43%)	14 (4%)	284 (90%)	28 (9%)	3 (0%)	2 (0%)	81 (26%)	87 (27%)	65 (21%)	54 (17%)	30 (9%)
2002	446	69 (10)	163 (37%)	27 (6%)	396 (89%)	44 (10%)	6 (0%)	0 (0%)	104 (23%)	115 (26%)	105 (24%)	75 (17%)	47 (11%)
2003	563	69 (10)	244 (43%)	35 (6%)	490 (87%)	66 (12%)	7 (0%)	0 (0%)	153 (27%)	140 (25%)	130 (23%)	99 (18%)	41 (7%)
2004	648	70 (10)	268 (41%)	36 (6%)	546 (84%)	89 (14%)	12 (0%)	1 (0%)	181 (28%)	166 (26%)	142 (22%)	100 (15%)	59 (9%)
2005	760	68 (11)	312 (41%)	51 (7%)	626 (82%)	114 (15%)	18 (0%)	2 (0%)	178 (23%)	190 (25%)	162 (21%)	153 (20%)	75 (10%)
2006	809	69 (11)	318 (39%)	48 (6%)	656 (81%)	131 (16%)	20 (0%)	2 (0%)	199 (25%)	210 (26%)	166 (21%)	150 (19%)	83 (10%)
2007	922	69 (11)	393 (43%)	56 (6%)	743 (81%)	154 (17%)	24 (0%)	1 (0%)	247 (27%)	240 (26%)	198 (21%)	167 (18%)	70 (8%)
2008	942	69 (11)	403 (43%)	52 (6%)	750 (80%)	158 (17%)	28 (0%)	6 (0%)	226 (24%)	227 (24%)	212 (23%)	181 (19%)	96 (10%)
2009	963	69 (11)	410 (43%)	46 (5%)	766 (80%)	166 (17%)	25 (0%)	6 (0%)	248 (26%)	259 (27%)	216 (22%)	166 (17%)	72 (7%)

For peer review only - http://bmjopen.bmj.com/site/about/guidelines.xhtml

2010	943	69 (11)	412 (44%)	57 (6%)	688 (73%)	222 (24%)	26 (0%)	7 (0%)	219 (23%)	250 (27%)	221 (23%)	154 (16%)	99 (10%)
2011	954	69 (10)	376 (39%)	64 (7%)	710 (74%)	191 (20%)	42 (0%)	11 (0%)	238 (25%)	256 (27%)	225 (24%)	170 (18%)	65 (7%)
2012	963	69 (10)	369 (38%)	66 (7%)	679 (71%)	224 (23%)	47 (0%)	13 (0%)	249 (26%)	226 (23%)	238 (25%)	166 (17%)	84 (9%)
2013	879	68 (11)	370 (42%)	43 (5%)	614 (70%)	213 (24%)	40 (0%)	12 (0%)	245 (28%)	215 (24%)	199 (23%)	145 (17%)	74 (8%)
2014	209	70 (10)	92 (44%)	14 (7%)	147 (70%)	52 (25%)	8 (0%)	2 (0%)	60 (29%)	46 (22%)	49 (23%)	35 (17%)	19 (9%)

For peer review only

Revision total knee replacement

Year of surgery	n	Age (mean (sd))	Gender: Male (n (%))	Rheumatoid arthritis (n (%))	RCS Charlson score (n (%)) 0	1	2	3+	IMD quintiles (n (%)) 1	2	3	4	5
1997	2	74 (1)	1 (50%)	0 (0%)	2 (100%)	0 (0%)	0 (0%)	0 (0%)	1 (50%)	1 (50%)	0 (0%)	0 (0%)	0 (0%)
1998	3	78 (2)	1 (33%)	2 (67%)	3 (100%)	0 (0%)	0 (0%)	0 (0%)	2 (67%)	0 (0%)	0 (0%)	1 (33%)	0 (0%)
1999	1	64 (-)	0 (0%)	1 (100%)	1 (100%)	0 (0%)	0 (0%)	0 (0%)	0 (0%)	0 (0%)	0 (0%)	1 (100%)	0 (0%)
2000	3	60 (19)	1 (33%)	3 (100%)	3 (100%)	0 (0%)	0 (0%)	0 (0%)	0 (0%)	1 (33%)	1 (33%)	0 (0%)	1 (33%)
2001	4	68 (13)	3 (75%)	0 (0%)	3 (75%)	1 (25%)	0 (0%)	0 (0%)	0 (0%)	2 (50%)	0 (0%)	1 (25%)	1 (25%)
2002	9	74 (9)	6 (67%)	1 (11%)	9 (100%)	0 (0%)	0 (0%)	0 (0%)	1 (11%)	6 (67%)	1 (11%)	1 (11%)	0 (0%)
2003	20	71 (11)	12 (60%)	3 (15%)	16 (80%)	4 (20%)	0 (0%)	0 (0%)	5 (25%)	2 (10%)	7 (35%)	5 (25%)	1 (5%)
2004	23	67 (10)	13 (57%)	4 (17%)	17 (74%)	4 (17%)	2 (0%)	0 (0%)	2 (9%)	8 (35%)	8 (35%)	4 (17%)	1 (4%)
2005	23	69 (11)	10 (43%)	4 (17%)	20 (87%)	3 (13%)	0 (0%)	0 (0%)	8 (35%)	4 (17%)	2 (9%)	8 (35%)	1 (4%)
2006	35	68 (9)	15 (43%)	6 (17%)	26 (74%)	8 (23%)	1 (0%)	0 (0%)	11 (31%)	8 (23%)	8 (23%)	6 (17%)	2 (6%)
2007	41	68 (10)	22 (54%)	4 (10%)	31 (76%)	5 (12%)	4 (1%)	1 (0%)	9 (22%)	5 (12%)	10 (24%)	13 (32%)	4 (10%)
2008	57	68 (10)	28 (49%)	9 (16%)	39 (68%)	17 (30%)	1 (0%)	0 (0%)	12 (21%)	12 (21%)	13 (23%)	11 (19%)	9 (16%)
2009	60	72 (10)	24 (40%)	7 (12%)	49 (82%)	10 (17%)	1 (0%)	0 (0%)	15 (25%)	17 (28%)	11 (18%)	14 (23%)	3 (5%)

For peer review only - http://bmjopen.bmj.com/site/about/guidelines.xhtml

2010	59	69 (10)	26 (44%)	8 (14%)	36 (61%)	20 (34%)	2 (0%)	1 (0%)	15 (25%)	15 (25%)	15 (25%)	10 (17%)	4 (7%)
2011	58	70 (10)	26 (45%)	6 (10%)	38 (66%)	18 (31%)	2 (0%)	0 (0%)	14 (24%)	16 (28%)	16 (28%)	9 (16%)	3 (5%)
2012	55	69 (10)	26 (47%)	3 (5%)	36 (65%)	15 (27%)	4 (1%)	0 (0%)	19 (35%)	12 (22%)	10 (18%)	12 (22%)	2 (4%)
2013	33	71 (9)	14 (42%)	3 (9%)	20 (61%)	9 (27%)	4 (1%)	0 (0%)	7 (21%)	9 (27%)	10 (30%)	6 (18%)	1 (3%)
2014	18	69 (9)	5 (28%)	1 (6%)	12 (67%)	5 (28%)	0 (0%)	1 (0%)	7 (39%)	2 (11%)	4 (22%)	3 (17%)	2 (11%)

For peer review only - http://bmjopen.bmj.com/site/about/guidelines.xhtml

Revision total hip replacement

					RCS Charlson score (n (%))				IMD quintiles (n (%))				
Year of surgery	n	Age (mean (sd))	Gender: Male (n (%))	Rheumatoid arthritis (n (%))	0	1	2	3+	1	2	3	4	5
1997	2	74 (11)	1 (50%)	1 (50%)	2 (100%)	0 (0%)	0 (0%)	0 (0%)	2 (100%)	0 (0%)	0 (0%)	0 (0%)	0 (0%)
1998	6	72 (8)	2 (33%)	0 (0%)	6 (100%)	0 (0%)	0 (0%)	0 (0%)	4 (67%)	2 (33%)	0 (0%)	0 (0%)	0 (0%)
1999	8	70 (14)	3 (38%)	1 (12%)	8 (100%)	0 (0%)	0 (0%)	0 (0%)	3 (38%)	3 (38%)	1 (12%)	1 (12%)	0 (0%)
2000	14	72 (11)	9 (64%)	2 (14%)	11 (79%)	3 (21%)	0 (0%)	0 (0%)	5 (36%)	1 (7%)	5 (36%)	1 (7%)	2 (14%)
2001	14	69 (10)	5 (36%)	2 (14%)	14 (100%)	0 (0%)	0 (0%)	0 (0%)	3 (21%)	2 (14%)	3 (21%)	5 (36%)	1 (7%)
2002	8	73 (11)	4 (50%)	2 (25%)	7 (88%)	1 (12%)	0 (0%)	0 (0%)	4 (50%)	1 (12%)	2 (25%)	0 (0%)	1 (12%)
2003	22	68 (11)	8 (36%)	7 (32%)	21 (95%)	1 (5%)	0 (0%)	0 (0%)	9 (41%)	5 (23%)	4 (18%)	2 (9%)	2 (9%)
2004	35	70 (10)	15 (43%)	6 (17%)	29 (83%)	6 (17%)	0 (0%)	0 (0%)	16 (46%)	7 (20%)	5 (14%)	3 (9%)	4 (11%)
2005	41	73 (10)	19 (46%)	7 (17%)	32 (78%)	9 (22%)	0 (0%)	0 (0%)	10 (24%)	9 (22%)	8 (20%)	8 (20%)	6 (15%)
2006	33	66 (12)	17 (52%)	5 (15%)	26 (79%)	3 (9%)	4 (1%)	0 (0%)	12 (36%)	8 (24%)	9 (27%)	1 (3%)	3 (9%)
2007	54	70 (10)	22 (41%)	5 (9%)	43 (80%)	10 (19%)	1 (0%)	0 (0%)	21 (39%)	16 (30%)	10 (19%)	3 (6%)	4 (7%)
2008	57	69 (11)	23 (40%)	8 (14%)	45 (79%)	10 (18%)	1 (0%)	1 (0%)	20 (35%)	12 (21%)	11 (19%)	10 (18%)	4 (7%)
2009	54	70 (13)	19 (35%)	9 (17%)	40 (74%)	12 (22%)	1 (0%)	1 (0%)	15 (28%)	18 (33%)	9 (17%)	9 (17%)	3 (6%)

For peer review only

2010	71	67 (12)	33 (46%)	7 (10%)	54 (76%)	14 (20%)	3 (0%)	0 (0%)	23 (32%)	20 (28%)	11 (15%)	9 (13%)	8 (11%)
2011	67	73 (11)	29 (43%)	4 (6%)	51 (76%)	7 (10%)	8 (1%)	1 (0%)	16 (24%)	14 (21%)	15 (22%)	12 (18%)	10 (15%)
2012	71	71 (9)	28 (39%)	14 (20%)	47 (66%)	15 (21%)	7 (1%)	2 (0%)	25 (35%)	15 (21%)	18 (25%)	10 (14%)	3 (4%)
2013	66	71 (9)	24 (36%)	10 (15%)	41 (62%)	17 (26%)	6 (1%)	2 (0%)	18 (27%)	20 (30%)	13 (20%)	8 (12%)	7 (11%)
2014	9	76 (14)	3 (33%)	1 (11%)	5 (56%)	3 (33%)	1 (0%)	0 (0%)	2 (22%)	2 (22%)	3 (33%)	1 (11%)	1 (11%)

For peer review only

For peer review only - http://bmjopen.bmj.com/site/about/guidelines.xhtml

LENGTH OF STAY BY YEAR OF SURGERY

Year	TKR		THR		TKR revision		THR revision	
	N	Mean (se)	N	Mean (se)	N	Mean (se)	N	Mean (se)
1997	45	18.89 (3.12)	78	14.28 (1.47)	2	12.5 (2.5)	3	27.33 (5.67)
1998	64	14.77 (0.78)	128	12.49 (0.42)	3	20 (2.08)	6	14.83 (1.08)
1999	114	12.79 (0.51)	195	12.1 (0.39)	1	18 (-)	8	18.75 (6.21)
2000	116	13.25 (1.09)	242	12.54 (0.64)	3	74 (59.03)	14	15 (1.79)
2001	170	12.13 (0.48)	319	11.97 (0.5)	4	15.25 (6.65)	14	21.86 (5.16)
2002	268	11.12 (0.42)	447	10.89 (0.26)	9	24 (7.11)	8	32.12 (12.92)
2003	403	10.32 (0.22)	561	10.3 (0.25)	21	21.29 (4.61)	23	22.48 (5.47)
2004	545	9.58 (0.3)	647	9.82 (0.29)	24	16.54 (3.61)	35	20.49 (3.42)
2005	604	9.11 (0.38)	761	9.33 (0.24)	22	19.64 (6.52)	40	21.48 (4.87)
2006	735	8.75 (0.27)	812	8.79 (0.22)	36	15.08 (3.35)	36	14.11 (2.87)
2007	913	8.1 (0.2)	923	8.25 (0.26)	41	13.41 (3.01)	58	15.28 (2.28)
2008	982	7.49 (0.24)	940	7.94 (0.3)	56	17.45 (4.1)	53	30.66 (5.38)
2009	1043	7.14 (0.21)	964	7.09 (0.18)	60	15.22 (2.74)	53	22.28 (4.33)
2010	1009	6.43 (0.11)	941	7.02 (0.17)	61	18.57 (5.55)	70	14.94 (3.05)
2011	1024	6.12 (0.1)	954	6.27 (0.13)	57	9.86 (1.35)	66	17.12 (2.55)
2012	1018	6.03 (0.16)	963	6.66 (0.3)	53	11.19 (2.03)	74	17.47 (3.05)
2013	967	5.89 (0.12)	877	5.83 (0.14)	35	15.4 (3.51)	63	11.87 (1.31)

For peer review only - http://bmjopen.bmj.com/site/about/guidelines.xhtml

2014	240	5.13 (0.15)	209	5.6 (0.3)	17	6.06 (0.8)	9	15.33 (4.78)

Number of study participants (N) with mean length of stay with standard error (se) by year. THR: total hip replacement, TKR: total knee replacement

For peer review only

HOSPITAL COSTS BY YEAR OF SURGERY

Year	Primary TKR		Primary THR		Revision TKR		Revision THR	
	N	Mean (se)	N	Mean (se)	N	Mean (se)	N	Mean (se)
1997	45	8516.45 (708.81)	78	7428.29 (359.68)	2	7562 (-)	3	9500.67 (2200.33)
1998	64	7590.65 (180.75)	128	7101.09 (98.33)	3	9677 (-)	6	7610.83 (362.58)
1999	114	7140.98 (128.49)	195	6921.47 (73.94)	1	8267 (-)	8	7244 (989.48)
2000	116	7168.41 (241.34)	242	6968.52 (103.49)	3	7562 (575.63)	14	7540.93 (500.08)
2001	170	6977.79 (100.8)	319	6839.25 (94.39)	4	6239.75 (1392.38)	14	8086.08 (670.15)
2002	268	6774.28 (89.49)	447	6669.12 (53.2)	9	9339.75 (1769.17)	8	10039.57 (2172.19)
2003	403	6590.4 (45.92)	561	6564.01 (54.2)	21	8544.84 (990.89)	23	8855 (907.28)
2004	545	6477.54 (68.11)	647	6417.76 (44.49)	24	7653.09 (493.94)	35	8442.61 (579.42)
2005	604	6393.04 (68.77)	761	6379.89 (53.06)	22	8306.64 (1423.54)	40	8688.02 (830.29)
2006	735	6401.5 (60.82)	812	6263 (40.8)	36	8026.77 (680.29)	36	7786.17 (468.81)
2007	913	6289.42 (42.25)	923	6155.92 (39.35)	41	8259.78 (595.51)	58	7942.64 (407.56)

For peer review only - http://bmjopen.bmj.com/site/about/guidelines.xhtml
For peer review only

2008	982	6194.13 (42.4)	940	6152.11 (58.67)	56	8249.2 (698.63)	53	10707.98 (989.17)
2009	1043	6202.38 (44.82)	964	5968.21 (24.92)	60	8056.98 (573.84)	53	8424.12 (614.65)
2010	1009	6107.99 (20.9)	941	6013.59 (31.66)	61	7829.78 (433.18)	70	8125.48 (608.8)
2011	1024	6108.19 (20.4)	954	5920.3 (24.32)	57	7130.88 (163.09)	66	8015.88 (383.55)
2012	1018	6136.84 (30.12)	963	6016.33 (47.13)	53	6906.06 (219.48)	74	8773.54 (596.93)
2013	967	6131.57 (22.07)	877	5900.26 (23.51)	35	8177.89 (737.29)	63	7523.76 (213.64)
2014	240	6053.24 (32.19)	209	5870.64 (50.52)	17	6761.94 (81.42)	9	7071.88 (615.47)

Number of study participants (N) with mean cost with standard error (se) by year. THR: total hip replacement, TKR: total knee replacement

For peer review only

PARTIAL EFFECT OF AGE ON LENGTH OF STAY AND COSTS

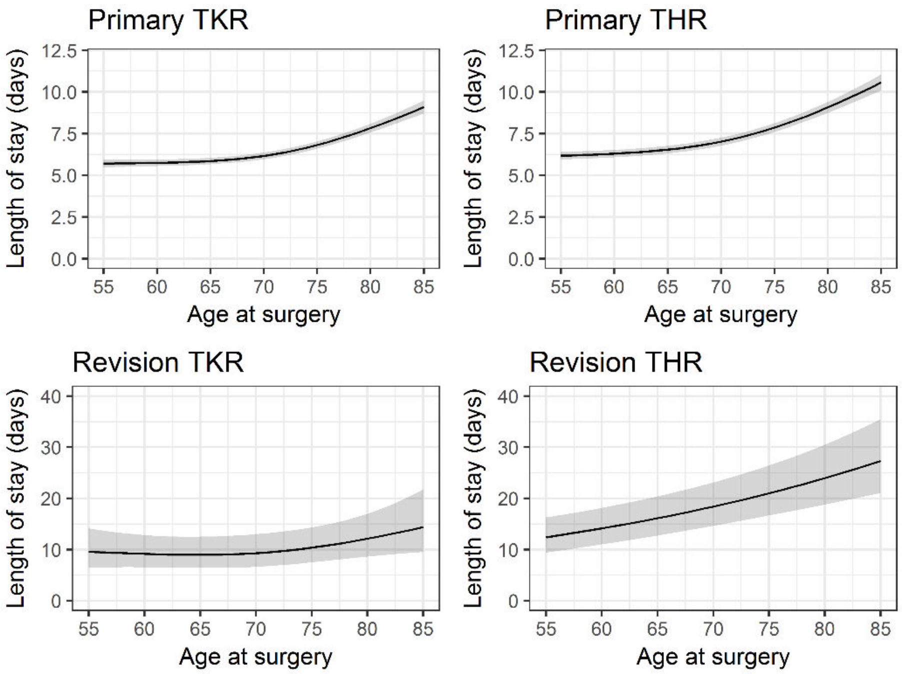

Partial effect of age at surgery on length of stay from multivariable regressions. Predicted length of stay by age while other explanatory variables are held fixed at their average (median if continuous and mode if categorical). TKR: total knee replacement, THR: total hip replacement.

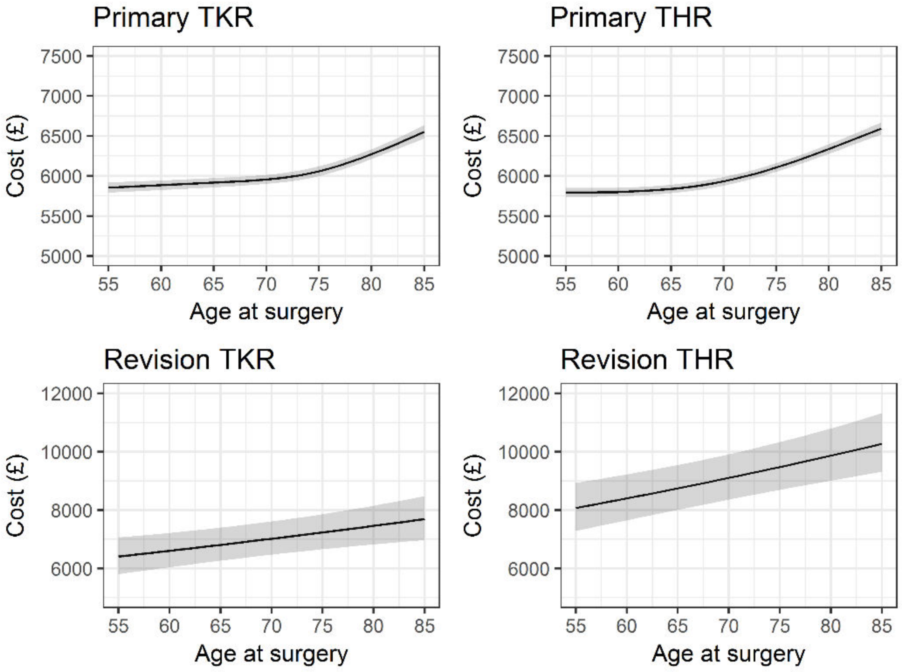

Partial effect of age at surgery on hospital costs (in 2017 GBP) from multivariable regressions. Predicted cost by age while other explanatory variables are held fixed at their average (median if continuous and mode if categorical). TKR: total knee replacement, THR: total hip replacement.

For peer review only - http://bmjopen.bmj.com/site/about/guidelines.xhtml

STROBE Statement—checklist of items that should be included in reports of observational studies

	Item No	Recommendation	Line No.
**Title and abstract**	1	(*a*) Indicate the study's design with a commonly used term in the title or the abstract	1
		(*b*) Provide in the abstract an informative and balanced summary of what was done and what was found	27-59
**Introduction**			
Background/rationale	2	Explain the scientific background and rationale for the investigation being reported	74-96
Objectives	3	State specific objectives, including any prespecified hypotheses	97-101
**Methods**			
Study design	4	Present key elements of study design early in the paper	103-111
Setting	5	Describe the setting, locations, and relevant dates, including periods of recruitment, exposure, follow-up, and data collection	122-137
Participants	6	(*a*) *Cohort study*—Give the eligibility criteria, and the sources and methods of selection of participants. Describe methods of follow-up<br>*Case-control study*—Give the eligibility criteria, and the sources and methods of case ascertainment and control selection. Give the rationale for the choice of cases and controls<br>*Cross-sectional study*—Give the eligibility criteria, and the sources and methods of selection of participants	153-167
		(*b*) *Cohort study*—For matched studies, give matching criteria and number of exposed and unexposed<br>*Case-control study*—For matched studies, give matching criteria and the number of controls per case	
Variables	7	Clearly define all outcomes, exposures, predictors, potential confounders, and effect modifiers. Give diagnostic criteria, if applicable	138-161
Data sources/ measurement	8*	For each variable of interest, give sources of data and details of methods of assessment (measurement). Describe comparability of assessment methods if there is more than one group	138-161
Bias	9	Describe any efforts to address potential sources of bias	
Study size	10	Explain how the study size was arrived at	
Quantitative variables	11	Explain how quantitative variables were handled in the analyses. If applicable, describe which groupings were chosen and why	171-177
Statistical methods	12	(*a*) Describe all statistical methods, including those used to control for confounding	163-182
		(*b*) Describe any methods used to examine subgroups and interactions	
		(*c*) Explain how missing data were addressed	183-186
		(*d*) *Cohort study*—If applicable, explain how loss to follow-up was addressed<br>*Case-control study*—If applicable, explain how matching of cases and controls was addressed<br>*Cross-sectional study*—If applicable, describe analytical methods taking	

For peer review only

		account of sampling strategy	
		(*e*) Describe any sensitivity analyses	
**Results**			
Participants	13*	(a) Report numbers of individuals at each stage of study—eg numbers potentially eligible, examined for eligibility, confirmed eligible, included in the study, completing follow-up, and analysed	Figure 1, 193-203
		(b) Give reasons for non-participation at each stage	Figure 1
		(c) Consider use of a flow diagram	Figure 1
Descriptive data	14*	(a) Give characteristics of study participants (eg demographic, clinical, social) and information on exposures and potential confounders	Table 1
		(b) Indicate number of participants with missing data for each variable of interest	Table 1
		(c) *Cohort study*—Summarise follow-up time (eg, average and total amount)	
Outcome data	15*	*Cohort study*—Report numbers of outcome events or summary measures over time	Table 1, Appendix
		Case-control study—Report numbers in each exposure category, or summary measures of exposure	
		Cross-sectional study—Report numbers of outcome events or summary measures	
Main results	16	(*a*) Give unadjusted estimates and, if applicable, confounder-adjusted estimates and their precision (eg, 95% confidence interval). Make clear which confounders were adjusted for and why they were included	Tables 2 and 3,
		(*b*) Report category boundaries when continuous variables were categorized	
		(*c*) If relevant, consider translating estimates of relative risk into absolute risk for a meaningful time period	205-210,
Other analyses	17	Report other analyses done—eg analyses of subgroups and interactions, and sensitivity analyses	
**Discussion**			
Key results	18	Summarise key results with reference to study objectives	243-265
Limitations	19	Discuss limitations of the study, taking into account sources of potential bias or imprecision. Discuss both direction and magnitude of any potential bias	267-282
Interpretation	20	Give a cautious overall interpretation of results considering objectives, limitations, multiplicity of analyses, results from similar studies, and other relevant evidence	289-332
Generalisability	21	Discuss the generalisability (external validity) of the study results	
**Other information**			
Funding	22	Give the source of funding and the role of the funders for the present study and, if applicable, for the original study on which the present article is based	340-344

*Give information separately for cases and controls in case-control studies and, if applicable, for exposed and unexposed groups in cohort and cross-sectional studies.

Note: An Explanation and Elaboration article discusses each checklist item and gives methodological background and published examples of transparent reporting. The STROBE checklist is best used in conjunction with this article (freely available on the Web sites of PLoS Medicine at http://www.plosmedicine.org/, Annals of Internal Medicine at

For peer review only

http://www.annals.org/, and Epidemiology at http://www.epidem.com/). Information on the STROBE Initiative is available at www.strobe-statement.org.

For peer review only

For peer review only - http://bmjopen.bmj.com/site/about/guidelines.xhtml
